# Allosteric couplings upon binding of RfaH to transcription elongation complexes

**DOI:** 10.1093/nar/gkac453

**Published:** 2022-06-07

**Authors:** José Alejandro Molina, Pablo Galaz-Davison, Elizabeth A Komives, Irina Artsimovitch, César A Ramírez-Sarmiento

**Affiliations:** Institute for Biological and Medical Engineering, Schools of Engineering, Medicine and Biological Sciences, Pontificia Universidad Católica de Chile, Santiago, Chile; ANID – Millennium Science Initiative Program – Millennium Institute for Integrative Biology (iBio), Santiago, Chile; Institute for Biological and Medical Engineering, Schools of Engineering, Medicine and Biological Sciences, Pontificia Universidad Católica de Chile, Santiago, Chile; ANID – Millennium Science Initiative Program – Millennium Institute for Integrative Biology (iBio), Santiago, Chile; Department of Chemistry and Biochemistry, University of California, San Diego, La Jolla, CA 92093, USA; Department of Microbiology and The Center for RNA Biology, The Ohio State University, Columbus, OH 43210, USA; Institute for Biological and Medical Engineering, Schools of Engineering, Medicine and Biological Sciences, Pontificia Universidad Católica de Chile, Santiago, Chile; ANID – Millennium Science Initiative Program – Millennium Institute for Integrative Biology (iBio), Santiago, Chile

## Abstract

In every domain of life, NusG-like proteins bind to the elongating RNA polymerase (RNAP) to support processive RNA synthesis and to couple transcription to ongoing cellular processes. Structures of factor-bound transcription elongation complexes (TECs) reveal similar contacts to RNAP, consistent with a shared mechanism of action. However, NusG homologs differ in their regulatory roles, modes of recruitment, and effects on RNA synthesis. Some of these differences could be due to conformational changes in RNAP and NusG-like proteins, which cannot be captured in static structures. Here, we employed hydrogen-deuterium exchange mass spectrometry to investigate changes in local and non-local structural dynamics of *Escherichia coli* NusG and its paralog RfaH, which have opposite effects on expression of xenogenes, upon binding to TEC. We found that NusG and RfaH regions that bind RNAP became solvent-protected in factor-bound TECs, whereas RNAP regions that interact with both factors showed opposite deuterium uptake changes when bound to NusG or RfaH. Additional changes far from the factor-binding site were observed only with RfaH. Our results provide insights into differences in structural dynamics exerted by NusG and RfaH during binding to TEC, which may explain their different functional outcomes and allosteric regulation of transcriptional pausing by RfaH.

## INTRODUCTION

Cellular RNA polymerases (RNAP) are multi-domain enzymes that transcribe the genomes in every domain of life, and their activities are elaborately controlled by a plethora of accessory proteins, among which NusG family is the only universally conserved group of transcription factors (1). NusG-like proteins have two key functions. First, their N-terminal domains (NTDs) bind to two mobile pincers of the elongating RNAP, the clamp and the lobe/protrusion domains (2–7), completing the circle around the nucleic acids to promote productive RNA synthesis (8). Second, their C-terminal domains (CTDs, one in prokaryotes or several in eukaryotes) bind to diverse cellular partners to coordinate transcription with other processes, such as translation in prokaryotes or splicing in eukaryotes (1).

The ubiquitous ‘anti-pausing’ activity of isolated NTDs (4,9,10) is commonly explained by their ability to act like processivity clamps (8). The RNAP pincers open to load the duplex DNA during initiation and close around the DNA upon the transition to elongation prior to processive RNA synthesis. By bridging the gap between the RNAP pincers, NusG homologs would guard against accidental opening of the clamp and premature termination. In addition to this local bridging effect, the NTD binding to RNAP could trigger an allosteric signal affecting distant elements in the enzyme, such as the catalytic center located tens of Angstroms away. Examples of allosteric modulators of RNA synthesis include nascent RNA hairpins (11), termination factor Rho (12,13), and antibiotics (14–16). Several RNAP inhibitors modulate clamp opening by binding to dynamic switch regions located at the base of the clamp domain (17,18).

**Figure 1.**
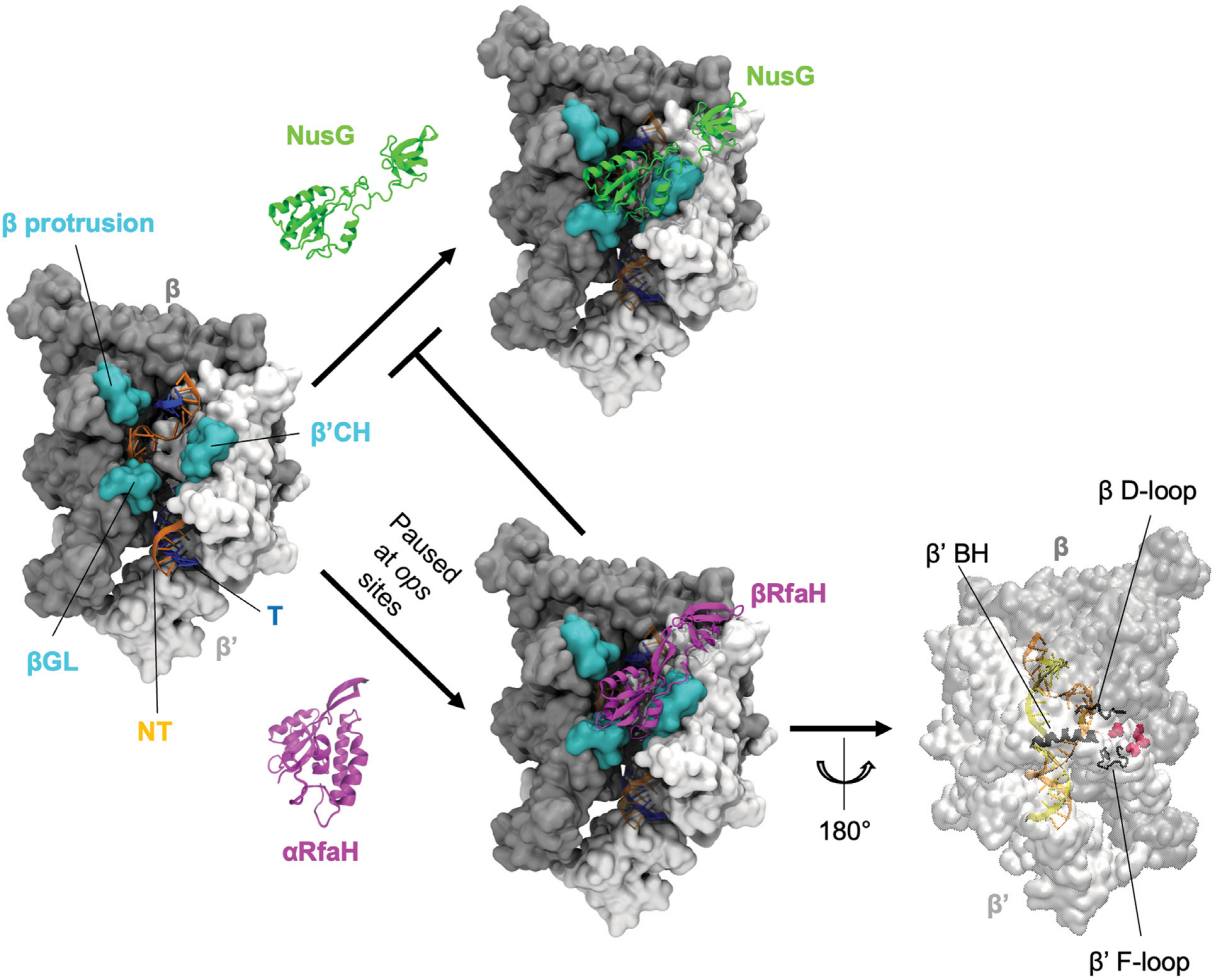
Structures of TECs bound to NusG and RfaH. RNAP (PDB 6c6s) is shown with β (gray) and β’ (white) subunits in surface representation and the DNA (blue for the template strand; orange for the non-template strand) in cartoon representation. Surfaces colored in cyan represent regions contacting both NusG and RfaH upon binding in the central cleft. Top: free and RNAP-bound NusG (NTD PDB 6c6u and CTD PDB 2jvv) is shown in green. Bottom: free autoinhibited αRfaH (PDB 5ond) and TEC-bound active βRfaH (PDB 6c6s) are shown in purple. A 180°-rotated view of RNAP shows several regions of its active center (black) that could be allosterically regulated by RfaH binding; the red spheres represent the binding site for CBR inhibitors. βGL: β gate loop; β’CH: β’ clamp helices; β’BH: β’ bridge helix; NT: non-template DNA strand; T: template DNA strand.

Here, we investigate interactions of two best-studied members of this family, *Escherichia coli* NusG and RfaH, with RNAP (Figure 1). NusG is a housekeeping factor that binds to RNAP transcribing most genes (19) and is essential for cellular viability. NusG NTD exhibits a mixed α/β fold connected via a flexible linker to a β-barrel CTD (10,20,21). The NTD inhibits RNAP backtracking, which can lead to arrest (22), while the CTD binds to ribosomal protein S10 to couple transcription to translation (23) or to Rho to terminate synthesis of antisense and foreign RNAs (24).

Expression of xenogeneic *E. coli* operons encoding biosynthesis of cell wall components, capsules, conjugation pili, and virulence factors, which are silenced by Rho and NusG, depends on a specialized paralog of NusG named RfaH (25). RfaH is recruited to RNAP paused at specific DNA sequences called *operon polarity suppressor* (*ops*) sites in the untranslated regions of these operons and blocks NusG from binding to RNAP, thus preventing Rho-dependent termination (Figure 1) (25).

Since RfaH opposes the essential function of NusG, its recruitment to RNAP must be tightly controlled. Sequence specificity of RfaH is determined by an unusually complex mechanism, which combines base-specific contacts of RfaH NTD with a short *ops* DNA hairpin, forming in the non-transcribed DNA strand exposed on the surface of RNAP, and a fold-switch between two native states (9,26,27). RfaH NTD shares the canonical fold of other NusG family proteins, but its CTD can be folded either as an autoinhibiting α-helical hairpin, which binds tightly to RfaH NTD (9), or as a NusG-like canonical β-barrel (28). The CTD dissociation from the NTD and concomitant refolding into the β-barrel is triggered by RfaH interactions with the TEC paused at the *ops* site (29) (Figure 1) and is, in turn, required for the productive RfaH NTD association with RNAP (30) and RfaH CTD binding to the ribosome for coupled translation (28).

While sequence-specific recruitment and fold-assisted autoinhibition are the key regulatory features of RfaH, *E. coli* NusG makes no specific contacts to DNA in the TEC and exists in only one, ‘active’ state (31). Structures of TECs bound to *E. coli* NusG and RfaH reveal similar contacts to RNAP: both factors bind above the central cleft surrounding the DNA through their NTDs (21,27,29,32), establishing contacts with the β subunit protrusion and gate loop (βGL) elements and with the β’ subunit clamp helices (β’CH), acting as a bridge between the two largest RNAP subunits (Figure 1). RfaH and NusG also stabilize the upstream edge of the transcription bubble, inhibiting RNAP backtracking (22,33).

Two subtle differences are apparent in their binding modes: NusG NTD makes more extensive contacts with the protrusion, which are blocked by RfaH-DNA interactions, and RfaH CTD could weakly bind the TEC, possibly to prevent its rebinding to the NTD prior to the ribosome recruitment (27) that could facilitate RfaH refolding towards the autoinhibited state (34). However, these differences do not explain why RfaH, but not NusG, suppresses transcription pausing and termination at some hairpin-dependent signals and precludes conformational changes that are associated with RNAP pausing, termed swiveling (27,30,35). A possible explanation may lie in altered dynamics of factor-bound transcription complexes, which cannot be captured by structural snapshots. Analysis of RfaH effects on RNA chain elongation and its interactions with RNAP variants with altered pausing properties led to a model in which RfaH, which binds 75 Å away from the RNAP catalytic center (Figure 1), modulates nucleotide addition allosterically (36). However, the structural changes that accompany RfaH and NusG binding to TEC remain largely unexplored.

Here, we used hydrogen-deuterium exchange mass spectrometry (HDXMS) to determine changes in local and non-local structural dynamics of *E. coli* NusG, RfaH and RNAP upon factor binding to *ops*-paused TEC. While specific RNAP-interacting regions in NusG and RfaH NTD become solvent-protected upon TEC binding, RfaH CTD shows increased deuterium uptake attributable not to its fold-switch behavior, but to local structural dynamics when folded as a β-barrel. For RNAP, opposite deuterium uptake changes are observed in regions interacting with NusG and RfaH. Strikingly, RNAP exhibits deuterium uptake changes far from the factor-binding site only upon binding of RfaH. Overall, our results provide insights of the differences in structural dynamics triggered by NusG and RfaH binding to TEC, as well as the role of allosteric regulation in RfaH action.

## MATERIALS AND METHODS

### Purification of RfaH, NusG and RNAP

*E. coli* RfaH was encoded in pIA777, a derivative of pET36b(+) containing NTD–TEV–CTD–[His6] (9). *E. coli* C41 (DE3) cells containing this plasmid were grown at 37°C in TB medium supplemented with kanamycin (50 μg/ml) until reaching an optical density at 600 nm (OD_600_) between 0.7 and 0.8, upon which protein overexpression was induced by adding 0.2 mM IPTG (US Biological, USA) at 20°C overnight. Cells were harvested by centrifugation (5,000 *g*, 30 min) and resuspended in buffer A (50 mM Tris–HCl pH 8.0, 400 mM NaCl, 5% glycerol) supplemented with 20 mM imidazole and 2 mM β-mercaptoethanol. Lysis was performed by sonication on ice, and the lysate was centrifuged at 15,000 *g* for 20 min. The protein-rich supernatant was loaded onto a HisTrap HP column (GE Healthcare, USA), washed, and then eluted using a linear gradient against buffer A supplemented with 250 mM imidazole. Finally, RfaH-containing fractions were pooled and dialyzed against buffer A supplemented with 2 mM β-mercaptoethanol before storing at -20°C.

*E. coli* NusG was encoded in pIA244, a derivate of pET33 containing [His6]-HMK-NusG (37). Protein overexpression and purification was performed identical to RfaH, except that the cell culture was induced at OD_600_ = 0.6–0.7 at 30°C for 3 h.

The isolated CTD of RfaH was purified as previously reported (38) from a [His6]–NTD–TEV–CTD RfaH encoded in pIA750, a derivative of pET28a (9).

*E. coli* RNAP was expressed from pVS10, which contains *rpoA-rpoB-rpoC* [His6] and *rpo*Z ORFs under control of T7 promoter (9). *E. coli* BL21 (DE3) cells containing this plasmid were grown at 37°C in LB medium until reaching an OD_600_ = 0.7, upon which overexpression was induced by adding 1 mM IPTG for 3 h. Cells were harvested by centrifugation (5,000 *g*, 30 min) and resuspended in buffer A^RNAP^ (50 mM Tris–HCl pH 6.9, 500 mM NaCl, 5% glycerol, 0.2 mM β-mercaptoethanol) supplemented with 20 mM imidazole and 0.2% Tween 20. Lysis was performed by sonication on ice and the lysate was centrifuged at 15,000 *g* for 20 min. The supernatant was loaded onto a HisTrap HP column, washed, and eluted using a linear gradient against buffer B^RNAP^ (buffer A^RNAP^ without NaCl and supplemented with 250 mM imidazole).

RNAP-containing fractions were pooled and loaded onto a Heparin HiTrap column (GE Healthcare, USA), washed, and then eluted with a linear gradient against buffer B^RNAP^ supplemented with 1.5 M NaCl. Peak fractions were pooled and dialyzed against buffer A^RNAP^. For ion-exchange chromatography, RNAP was loaded onto a MonoQ column (GE Healthcare, USA), washed, and eluted as described for heparin-affinity chromatography. Finally, pooled RNAP was dialyzed against buffer containing 10 mM Tris–HCl pH 7.5, 50% glycerol, 100 mM NaCl, 0.1 mM EDTA and 0.1 mM DTT and stored at -20°C.

### Assembly of *ops*-TEC

Assembly of free TEC paused at *ops* site was based on previous publications using appropriately designed oligonucleotides (see Supplementary Information*)* (27). First, the template DNA:RNA hybrid was formed by mixing each oligonucleotide at a final concentration of 4 μM in buffer TB-40 (20 mM Tris–HCl pH 7.9, 40 mM KCl, 5.0 mM MgCl_2_, 1.0 mM β-mercaptoethanol, 6.0% glycerol). Annealing was performed by incubating the oligonucleotides in a thermocycler according to the following protocol: 45°C for 5 min; 42°C, 39°C, 33°C, 30°C and 27°C for 2 min each; 25°C for 10 min. Next, RNAP was added equimolarly to previously annealed DNA:RNA hybrid in the same buffer and incubated at 25°C for 10 min. Finally, the non-template DNA was added at 2-fold molar excess over DNA:RNA:RNAP complex in the same buffer and incubated at 25°C for 10 min.

NusG- and RfaH-bound TEC were obtained by mixing free TEC at a molar ratio 1:1 for NusG and 3:1 for RfaH in buffer TB-40 and incubated at 25°C for 30 min. Complex formation was assessed by visual inspection in agarose gel at 1% (Supplementary Figure S1). Free and factor-bound samples were stored at -20°C.

### Hydrogen–deuterium exchange mass spectrometry

HDXMS was performed using a Synapt G2Si system with H/DX technology (Waters Corp, Milford, MA, USA) as in our previous work (38). Briefly, 5 μL protein aliquots at 1–3 μM protein concentration were allowed to exchange at 25°C for 0, 0.5, 1 or 2 min in 55 μL of deuterated buffer TB-40. Reactions were quenched by mixing with an equal volume of a solution containing 2 M guanidine HCl and 1% formic acid (pH 2.66) at 1°C for 2 min. Quenched samples were subjected to online proteolysis and peptide separation by injection into a custom-built pepsin-agarose column (Thermo Fischer Scientific, Waltham, MA, USA) followed by ultra-performance liquid chromatography at 1°C. The eluting peptides from the analytical column were directly electrosprayed into a Synapt G2-Si quadrupole time-of-flight (TOF) mass spectrometer (Waters Corp) set to Mobility-MS^E^-ESI + mode for initial peptide identification and to Mobility-TOF-ESI + mode to collect H/DX data.

Deuterium uptake was determined by calculating the shift in the centroids of the mass envelopes for each peptide compared with the nondeuterated controls, using the DynamX 3.0 software (Waters Corp). Back-exchange was corrected using the software DECA v1.14 (39), available in https://github.com/komiveslab/DECA, using the peptide 121–129 of TEC-bound RfaH as template (Supplementary Table S1). Also, the difference in deuterium uptake of overlapping peptides was used for calculating the incorporation of overhanging regions when the difference in mass exceeded 5 times its uncertainty (see Supplementary Information*)*. The maximum deuterium uptake per peptide was employed for calculating the difference in deuteron incorporation for local regions of RfaH, NusG and TEC in the free and bound forms (Supplementary Tables S1-S7), which was obtained by fitting the back-exchange corrected data to a single exponential (Supplementary Tables S8-S14).

### Molecular dynamics and estimation of hydrogen–deuterium exchange

Explicit-solvent molecular dynamics (MD) simulations were carried out for RfaH, NusG and RfaH-bound TEC. Missing interdomain loop residues 98–117 in the crystal structure of RfaH (PDB 5ond) were added using Modeller (40). Full-length NusG was modeled using Colabfold (41), a Google Colaboratory implementation of AlphaFold2 (42). RfaH-bound TEC was obtained from the cryo-EM structure (PDB 6c6s).

Simulations were run in Amber20 suite (43), using the AMBERff14SB force field (44) for proteins, OL15 (45) for DNA, and OL3 (46) for RNA. All simulation systems were filled in an octahedral geometry with 1.3 nm padding of TIP3P water molecules and neutralized with counterions. Each system was energy-minimized, and then temperature- and pressure-equilibrated using a Langevin thermostat at a constant temperature of 298.15 K and a Berendsen barostat at a constant pressure of 1 atm. MD production runs of 100 ns were obtained for each system, using a timestep of 2 fs along with the SHAKE algorithm (47) to constrain hydrogen-containing bonds and particle mesh Ewald method (48) for long range electrostatics.

Computational estimation of backbone amide HDX from the MD production runs was achieved based on a previously described approach (49). First, a custom Tcl script for VMD (50) was employed to determine the formation of hydrogen bonds by each backbone amide with either the protein or the surrounding water molecules, based on which their exchange protection factors (PF) are predicted using a logistics growth function (49). The intrinsic exchange rate (*k*_int_) for each residue was obtained using SPHERE (https://protocol.fccc.edu/research/labs/roder/sphere/sphere.html) and used alongside their predicted PF to calculate their deuteron incorporation using the equation described in Supplementary Figure S2.

## RESULTS

### The NTD in both RfaH and NusG becomes protected upon TEC binding

Members of the NusG family have an NTD with a conserved α/β fold. Despite their low sequence identity (21%), both RfaH and NusG bind to the same site on the TEC via similar - but not identical - contacts between their NTD and the β and β’ RNAP subunits (27). Notwithstanding these structural similarities, *E. coli* NusG and RfaH exert different effects on RNA chain elongation and termination (30,35,51). Thus, we decided to inspect changes in local structural dynamics upon formation of the NusG- and RfaH-bound TECs that could contribute to their functional differences.

**Figure 2.**
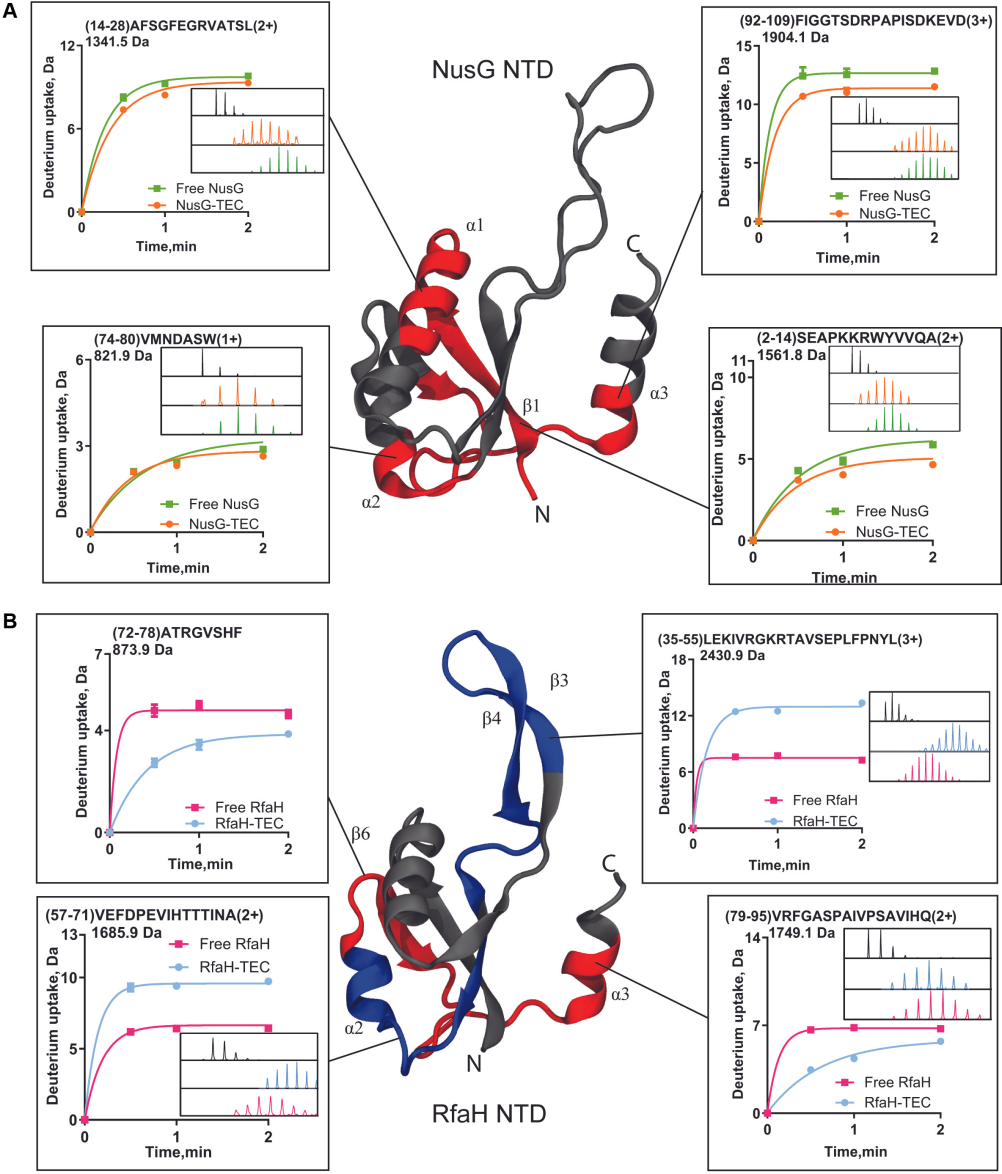
Changes in deuterium uptake observed with the NTD of NusG and RfaH upon binding to the *ops*-TEC. The corresponding regions are mapped on the structures of NusG NTD (PDB 6c6u) (**A**) and RfaH NTD (PDB 6c6s) (**B**) and shown in red for peptides with lower uptake and blue for higher uptake upon TEC binding. Each plot shows the deuterium uptake kinetics of each peptide identified by MS or obtained after subtraction between two or more peptides, with the inset displaying the mass spectra of the nondeuterated sample (black) and after 2 min of exchange in deuterated buffer in the free and TEC-bound conditions. For simplicity, only two peptides with the highest increase in exchange are shown for RfaH but all others are listed in Supplementary Table S1.

To shed light on the differences in local structural dynamics between RfaH and NusG upon TEC binding, we performed HDXMS experiments on the free and TEC-bound factors. Given the structural data available, we first started by analyzing the changes in deuterium uptake between the NTDs of RfaH and NusG upon TEC binding. For proper comparisons, we selected all RfaH and NusG peptides identified in both free and TEC-bound conditions, resulting in 10 peptides for NusG NTD (residues 2–133) and 11 peptides for RfaH NTD (residues 1–107), which cover structurally similar regions in both proteins. Then, we calculated the maximum deuterium uptake for each peptide in the free and TEC-bound states (Figure 2) and the difference in deuterium uptake for RfaH and NusG between the free and TEC-bound states (Supplementary Tables S1 and S2).

NusG NTD showed a significant decrease (Supplementary Figure S3) in deuterium uptake upon TEC binding in 4 out of 10 peptides, comprising 35% of NTD sequence (Figure 2A). These regions comprised strand β1 (residues 2–14), helix α1 (residues 14–28), α2 (residues 74–80) and the end of strand β4 and helix α3 (residues 92–109). In contrast, RfaH NTD only exhibited lower uptake for residues 72–78 and 79–95 upon TEC binding, corresponding to 0.9 deuterons in both cases (Figure 2B and Supplementary Figure S3). These regions comprise strand β6 and helix α3 and only cover 24% of the NTD sequence.

These results suggest that both RfaH and NusG NTD become solvent-protected in these regions upon binding to TEC. A structural alignment using STAMP (52) on the solved cryo-EM structures of these complexes show that RfaH residues 79–95 match NusG residues 92–109 and that both proteins make direct contacts with the RNAP central cleft through these regions (21,27), thus explaining the decrease in deuterium uptake observed for these peptides.

Remarkably, almost all remaining residues in RfaH NTD, represented by 7 out of 11 identified peptides, significatively exchanged more deuterons when bound to TEC (Figure 2B). Among them, residues 35–55 exchanged around five more deuterons upon TEC binding. This region belongs to a β-hairpin (β3-β4), which makes up the interface between NTD and αCTD in the autoinhibited RfaH but is extensively unstructured in NusG (9). Experimental and computational studies have proposed that interdomain contacts, including those formed by residue E48 located in this β-hairpin, are key in controlling RfaH fold-switching (28,38,53,54). Another region with a notable increase corresponds to the flexible interdomain loop (residues 96–107), which incorporated almost 6 deuterons, thus suggesting that the loop in the α-folded RfaH is more restrained than when bound to the TEC. Finally, RfaH residues 57–71 in helix α3 also showed an increase in ∼3 deuterons upon complex formation. Residues 65–68 in this peptide comprise the HTTT motif, whose interactions with βGL are required for RfaH anti-pausing activity but not for binding to TEC (7,55). NusG also interacts with βGL (residues 79–82), but these interactions may not be strictly necessary for its function (22,56).

Since RfaH undergoes a dramatic structural change enabled by the CTD dissociation from the NTD (28), it is not obvious how much of the observed local deuteration of RfaH NTD is due to the lost contacts with the CTD versus TEC binding. Therefore, we explored the separate contributions of both events to the observed deuteron uptake for RfaH NTD using MD simulations and backbone amide hydrogen bonding analysis (Supplementary Figure S2A), which enables one to predict the extent of deuteration of different parts of the protein (49).

For these analyses, we assumed that RfaH must reach an ‘open state’, in which the CTD is dissociated from the NTD, prior to establishing contacts observed in the RfaH-TEC complex. Based on this assumption, we performed MD simulations for full-length RfaH (‘closed state’), its isolated NTD (‘open state’), and TEC-bound RfaH, as well as for NusG as a control (Supplementary Figures S2B and S2C). From these simulations, the PF of each amide was calculated and employed alongside the intrinsic rate of exchange of each residue to predict their deuteron incorporation and compare it against the deuteration extent for the peptide identified by HDXMS.

**Figure 3.**
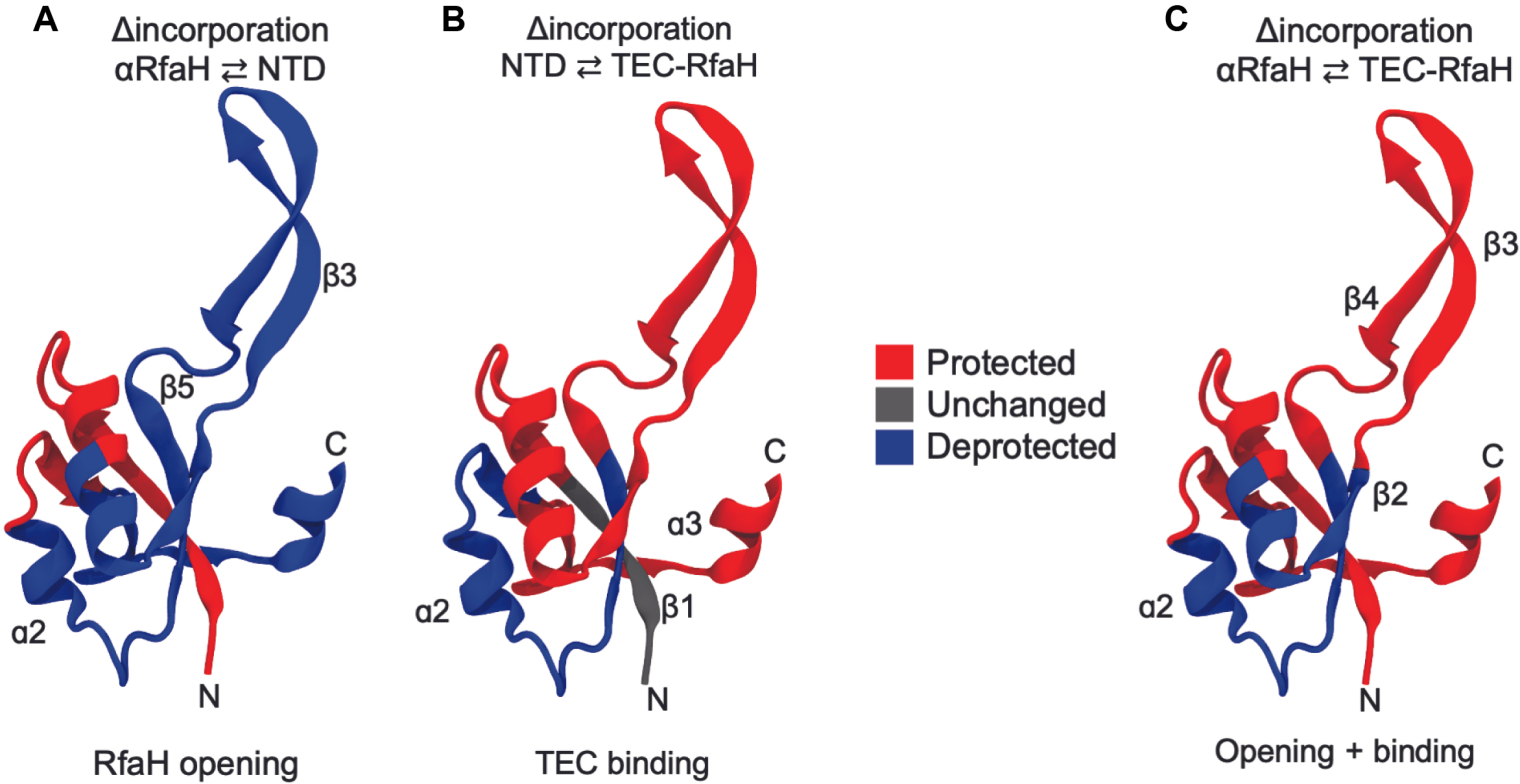
Computationally predicted changes in deuterium uptake for RfaH NTD due to domain dissociation and TEC binding. Deuteron incorporation was predicted from 100 ns of explicit-solvent MD simulations to explore the changes due to RfaH opening (**A**) or NTD binding to the TEC (**B**). The change in deuteron incorporation for the complete process of opening and binding was calculated (**C**) for comparison against the experimental HDXMS.

The results from these computational predictions are shown in Figure 3, where the changes in deuteron incorporation between full-length RfaH and the isolated NTD describe the effects of RfaH opening (Figure 3A) and the changes in uptake between the isolated NTD and the TEC-bound RfaH describe the effect of TEC binding (Figure 3B).

These MD simulations suggest that RfaH opening exposes most of the NTD amides for exchange (Figure 3A), except for the first 19 residues, which exhibit a predicted decrease in incorporation of 1.5 deuterons, and residues 72–78 which increase incorporation by 0.5 deuterons. In contrast, it is predicted that upon NTD binding to the TEC most residues become exchange-protected, except for those comprising strands β5 and β6 and helix α2 (Figure 3B). The predicted deuteration of the NTD for the overall process of RfaH opening and binding to the TEC (Figure 3C) largely resembles the experimentally determined change in deuterium uptake observed by HDXMS (Figure 2B), except for the β-hairpin that becomes more deuterated when bound to the TEC but is predicted to exchange less in our simulations. This discrepancy is likely due to the binding of this element to the β-barrel RfaH CTD in the cryo-EM structure employed as the starting configuration (PDB 6c6s), thus impeding its hydrogen bonding with water molecules in our simulations. These results further confirm that RfaH NTD residues covering strand β2 and helix α2 become more solvent accessible or more dynamic upon binding to the TEC.

### The CTD of RfaH and NusG show opposite exchange behaviors upon TEC binding

We then analyzed the changes in deuterium uptake of the CTD of full-length NusG and RfaH upon TEC binding. This domain constitutes the most significant structural divergence between the paralogs, as the CTD of RfaH fold-switches between two native states, one of which is identical to the canonical β barrel of NusG CTD (28), in the course of binding to the TEC (29).

**Figure 4.**
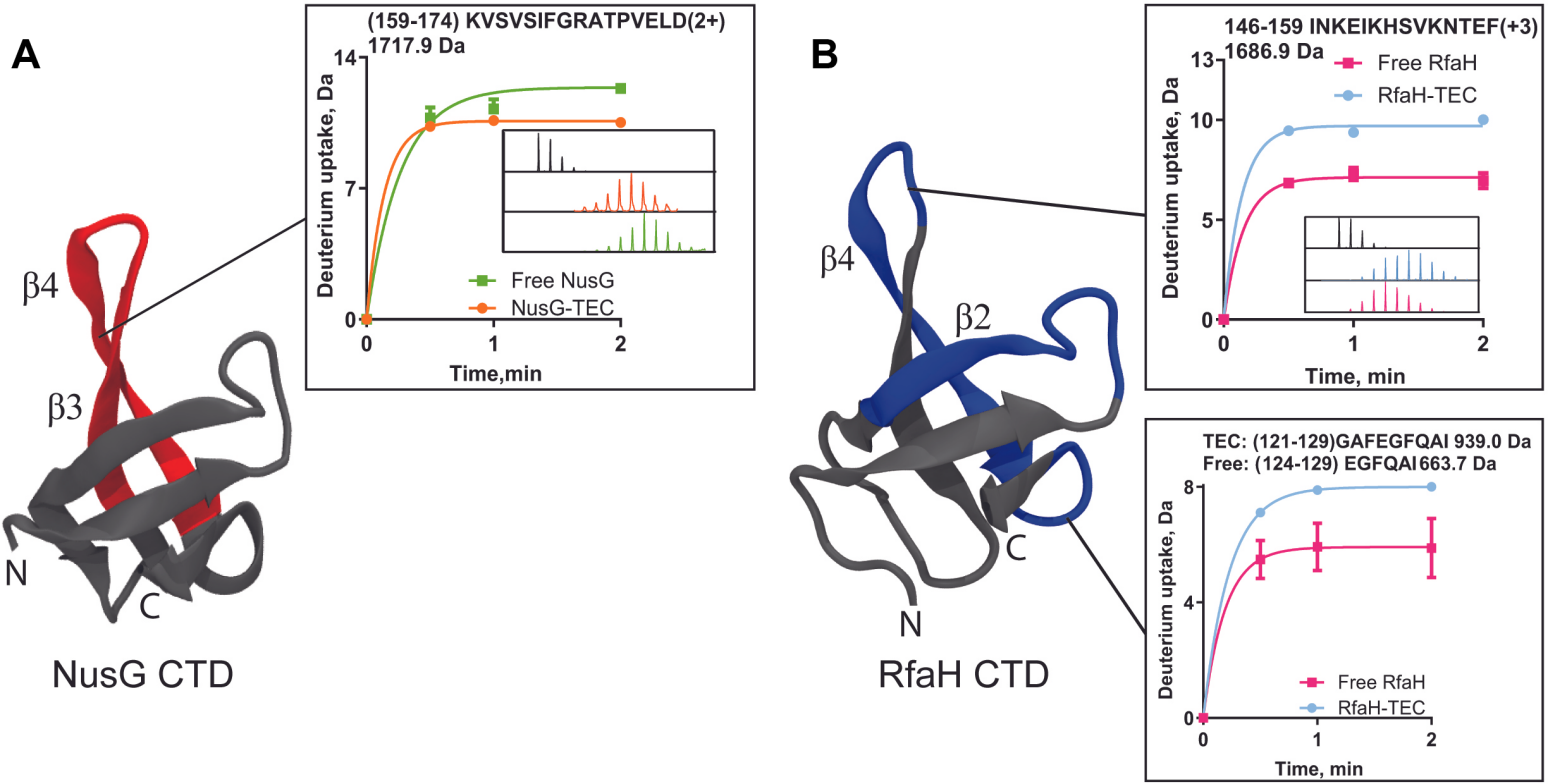
Changes in deuterium uptake on the CTDs of NusG and RfaH upon binding to the *ops*-TEC. The corresponding regions are mapped on the structures of NusG CTD (PDB 2jvv) (**A**) and RfaH CTD (PDB 6c6s) (**B**) and are shown in red for peptides with lower uptake and in blue for higher uptake upon TEC binding. Each plot shows the deuterium uptake kinetics of each peptide identified by MS or obtained after subtraction between two or more peptides, with the inset displaying the mass spectra of the nondeuterated sample (black) and after 2 min of exchange in deuterated buffer in the free and TEC-bound conditions.

We identified six peptides for NusG CTD, covering residues 134–181. For this domain, only peptide 159–174, comprising strands β3-β4, showed a significant decrease (1.8 deuteron) in incorporation after binding to the TEC (Figure 4A, Supplementary Table S2 and Figure S3). All other peptides did not show a significant decrease in deuterium uptake that would be indicative of potential interactions with RNAP. In fact, NusG CTD interacts with other macromolecules, such as the termination factor Rho or the ribosomal protein S10 (23,57). However, it has been reported that this domain does not appear to make productive contacts to RNAP (32) and the CTD was not resolved in the cryo-EM structures of NusG complexed with *E. coli* TECs (13,27), suggesting that it is highly mobile. Also, analyses of coupled transcription-translation complexes reveal that NusG NTD binds RNAP while NusG CTD interacts exclusively with the ribosome (21,32,58). Nonetheless, it is noteworthy that changes in deuterium uptake are consistent with recent NMR studies on NusG CTD, which showed that in solution this domain is mostly rigid, with the exception of the loop between β3 and β4 (59).

When RfaH is recruited to RNAP paused at the *ops* site, its CTD separates from its NTD and refolds from an α-hairpin into the canonical β-barrel of NusG family (28,29). Moreover, its isolated CTD in solution has a β-barrel structure (28). When calculating the differences in uptake of RfaH CTD between free and TEC-bound factors, all the identified peptides showed a sharp increase in uptake, between one and eight deuterons, approximately (Supplementary Table S1). To deduce the effect of RfaH fold-switching in the exchange, we calculated the differences in deuterium uptake between TEC-bound RfaH and the isolated RfaH CTD (Supplementary Table S3).

We identified 6 contiguous peptides in the isolated RfaH CTD (covering residues 117–159) and five peptides in the CTD of TEC-bound RfaH (covering residues 108–159) (Supplementary Tables S1 and S3). RfaH CTD showed a higher uptake upon TEC binding in residues 121–129 and 146–159, with increases greater than 2 deuterons (Figure 4B and Supplementary Figure S3). These results indicate that both fold-switching and TEC binding cause changes in local structural dynamics of RfaH CTD.

The local structural dynamics of RfaH and NusG ascertained by HDXMS show both similarities and differences in many regions related to their function. On the one hand, regions that mediate interactions of these proteins with RNAP showed lower uptake upon TEC binding. On the other hand, regions implicated in the metamorphic behavior of RfaH, namely the NTD β-hairpin and the CTD, showed an opposite effect when compared with NusG.

To determine if the observed deuterium uptake is consistent with the molecular information provided by solved structures of free and TEC-bound RfaH and NusG, we compared the deuterium uptake from our HDXMS experiments with the solvent accessible surface area (SASA) calculated from the available solved structures using GetArea (60). For factor-bound complexes, we employed the cryo-EM structures of TEC-NusG and TEC-RfaH. For free NusG, we completed the NTD from the cryo-EM structure (lacking residues 49–62) by adding the NMR-solved isolated CTD (residues 134–181) using Coot (61). Since NusG CTD is not modeled in the TEC-bound structure, we employed the SASA calculated for the free CTD.

**Figure 5.**
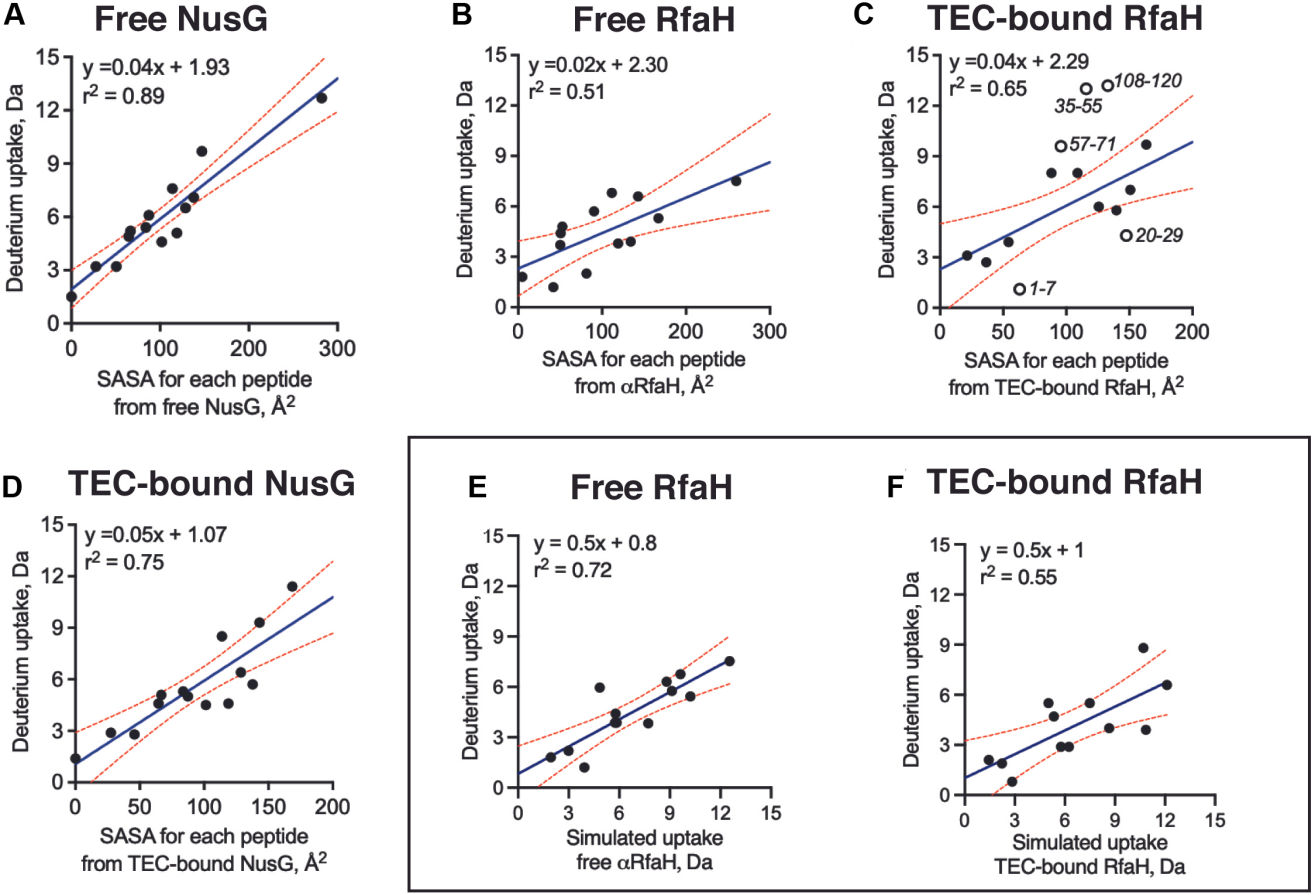
Correlation between experimental deuterium uptake and computationally determined SASA and deuterium uptake for free and TEC-bound NusG and RfaH. Plots A–D show the correlation between maximum deuterium uptake for each peptide analyzed by HDXMS and the backbone SASA calculated from the solved structures in the free and TEC-bound states. SASA for free (**A**) and TEC-bound (**D**) NusG was calculated from the available NTD (PDB 6c6u) and CTD structures (PDB 2jvv). SASA for free (**B**) RfaH was calculated from crystallographic structure of free RfaH (PDB 5ond), whereas SASA for TEC-bound RfaH was calculated from cryo-EM structure (**C**, PDB 6c6s). Open circles correspond to peptides removed before data fitting (whole data fitting *r*^2^ = 0.31). The rectangular box highlights the correlation between experimental deuterium uptake for all identified peptides by HDXMS and the predicted deuteron incorporation obtained from MD simulations for free RfaH (**E**) and TEC-bound RfaH (**F**).

Except for the peptides in modelled loops (residues 49–70 and 110–133), both free and TEC-bound NusG showed good correlation between deuterium uptake and backbone SASA for all peptides analyzed (free NusG, *r*^2^ = 0.89; TEC-bound NusG, *r*^2^ = 0.75), indicating that the changes in deuterium uptake in free and TEC-bound NusG are consistent with the solved structures and are mostly explained by changes in solvent accessibility upon complex formation (Figure 5A and B).

In free αRfaH (a crystallographic structure lacking residues 98–117 that belong to the interdomain linker), the extent of exchange showed a modest correlation with backbone SASA (*r*^2^ = 0.51, Figure 5B). However, TEC-bound RfaH showed poor correlation (*r*^2^ = 0.31), which only improved by removing 5 poorly correlated peptides within the NTD (*r*^2^ = 0.63, Figure 5C). The removed peptides cover regions 1–7, 20–29, 35–55, 57–71 and 108–120. Residues 35–55 correspond to the NTD β-hairpin that shows higher uptake upon binding to TEC (Figure 2). The β-hairpin is a mobile domain in RfaH (29), which could explain the higher uptake observed and its poor correlation with the calculated SASA. A similar behavior was observed for peptide 20–29, with residues such as H20 related to DNA binding; peptide 57–71, a region implicated in anti-pausing activity; and peptide 108–120, a flexible linker connecting both domains (55). Remarkably, the CTD peptides showed good correlation between deuterium uptake and backbone SASA.

It is important to consider that for globular proteins, the amide exchange can be also affected by other constraints such as flexibility, distance of the amide from the surface, and even side-chain influence (62). Indeed, a better correlation with the experimental data is observed when comparing all peptides against the predicted deuteron incorporation estimated from our simulations (Figure 5E, F) on αRfaH (*r*^2^ = 0.72) and on TEC-bound RfaH (*r*^2^ = 0.55). By contrast, the strong correlation between the computed exchange and the experimental data for NusG yields an *r*^2^ = 0.88 (Supplementary Figure S2), thus indicating that most exchange data can be explained solely by its structure.

Altogether, these data suggest that the HDXMS results are consistent with the structural information available for NusG, while RfaH shows a more complex behavior that cannot be explained solely by the solvent accessibility observed in the solved structures of its free and TEC-bound states and that is partly explained by the local structural dynamics of RfaH ascertained by MD simulations.

### RfaH and NusG exert local and allosteric effects on RNAP structural dynamics

We also analyzed changes in deuterium uptake in RNAP upon NusG and RfaH binding, mainly focusing on RNAP subunits β and β’ that form the active site in the polymerase. We identified 49 peptides for the β subunit in TEC-NusG (48.5% coverage, Supplementary Table S4) and 45 peptides in TEC-RfaH (41% coverage, Supplementary Table S5), while 28 peptides were identified for β’ subunit in TEC-NusG (27.2% coverage, Supplementary Table S6) and 38 peptides in TEC-RfaH (32.1% coverage, Supplementary Table S7). We then searched for peptides with significant differences in uptake between factor-bound and free TEC, based both on the change in incorporated deuterons and the experimental uncertainty (standard deviation) for each peptide (Supplementary Information and Supplementary Figure S3).

**Figure 6.**
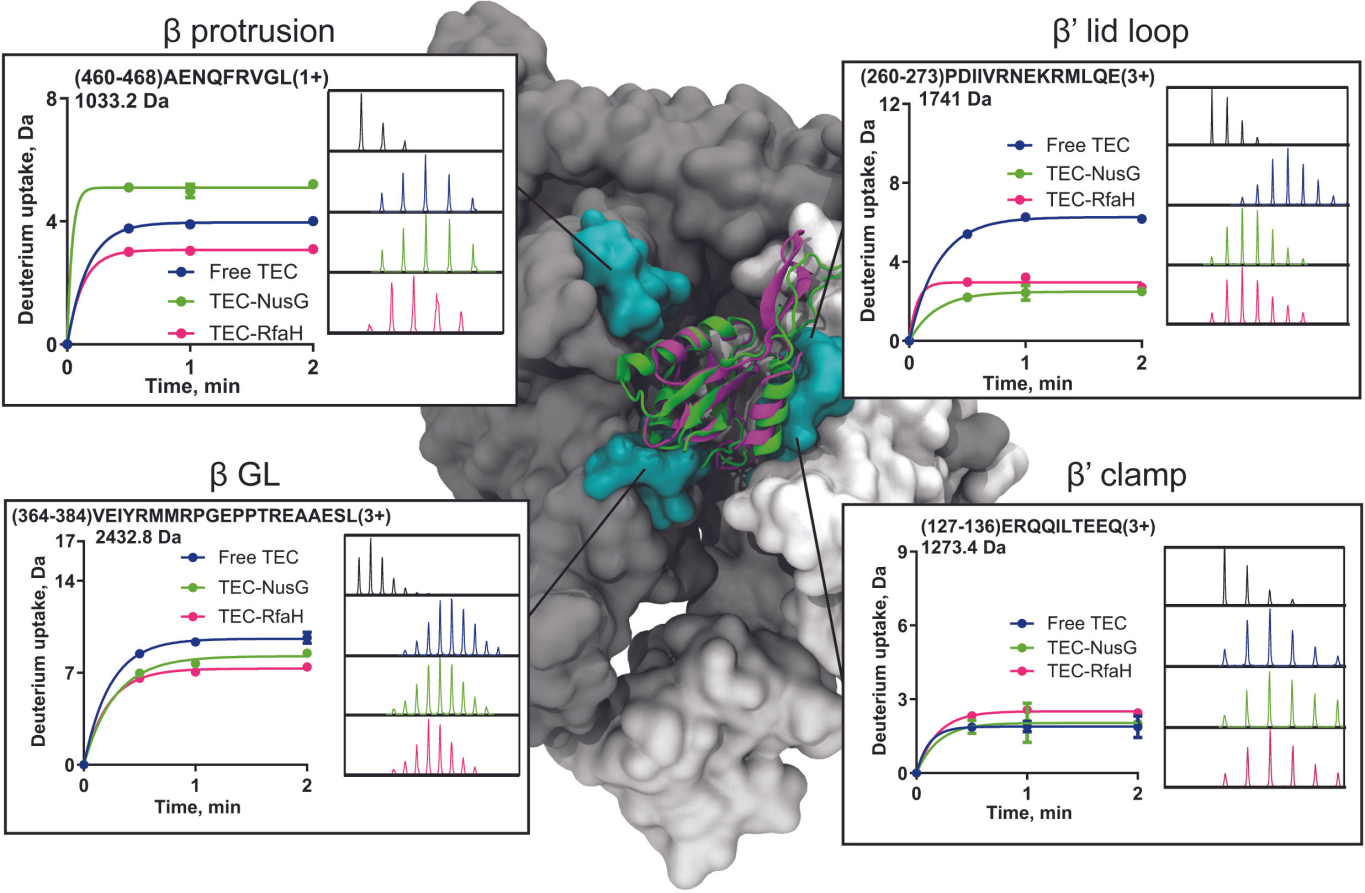
Changes in RNAP deuterium uptake upon factor binding. Surfaces colored in cyan represents regions contacting the factors in the central cleft formed by β (gray surface, left plots) and β’ (white surface, right plots). RfaH and NusG are shown in cartoon representation in magenta and green, respectively. Each plot shows the deuterium uptake for factor-bound and free TEC over time, with the inset displaying the mass spectra of the nondeuterated sample (black) and after 2 min of exchange in deuterated buffer in the free (blue), NusG-bound (green) and RfaH-bound (pink) conditions.

RNAP interacts similarly with NusG and RfaH, surrounding the NTD with the β protrusion/GL and the β’CH (21,27,32). However, while residues 18–34 of NusG interact with the protrusion (an α-hairpin covering residues 460–514), the corresponding region of RfaH (residues 13–24) interacts with the *ops* DNA hairpin instead (27). Consistently, we observed opposite effects in the protrusion upon NusG or RfaH binding (Figure 6). For the first helix (residue 460–468; Figure 6 and Supplementary Figure S3), NusG binding led to an increase in uptake of ∼1 deuteron while RfaH caused a decrease of ∼1 deuteron. Regarding the rest of this hairpin, we were unable to analyze peptides from the tip and residues 485–514 did not show significant differences.

By contrast, RfaH and NusG make similar contacts to RNAP near the upstream edge of the transcription bubble, and both proteins inhibit RNAP backtracking by promoting DNA strand reannealing (22,33). The β’ lid loop (residues 251–263) has been shown to act in concert with NusG to stabilize the upstream DNA (22) and is expected to have a similar effect on RfaH. Consistently, we observed that binding of both factors to TEC led to a similar decrease in deuterium uptake for residues 260–273 (NusG, 3.8 deuterons; RfaH, 3.3 deuterons; Figure 6; Supplementary Tables S6 and S7 and Supplementary Figure S3).

In the case of βGL, a conserved domain implicated in anti-pausing and DNA chaperoning functions of RfaH (7,33,63), binding of both NusG and RfaH led to a decrease in uptake of ∼1.3 and 2.3 deuterons for residues 364–384, respectively (Figure 6 and Supplementary Figure S3). It has been proposed that unrestrained motions of βGL would favor RNAP pausing and that RfaH binding could reduce βGL mobility to inhibit pausing prior termination (7,55,63). Changes in deuterium uptake support this antitermination mechanism, even for NusG (64).

The β’CH domain (residues 265–307) is the main binding determinant for NusG and RfaH (27,55). The β’CH is part of the β’ clamp, a large mobile domain of RNAP whose movements control every step of the transcription cycle. Clamp opening is thought to promote pausing and termination (13,65), and, similarly to its proposed effect on βGL, RfaH could restrict the β’ clamp mobility to inhibit pausing (7). Unfortunately, we were only able to observe subtle significant differences in deuteron incorporation for this domain in residues 127–136 (Figure 6), a DNA-interacting region located downstream in the nucleic acid channel, and only upon RfaH binding (Supplementary Tables S6 and S7). Thus, our data provides limited information on the direct effect of RfaH/NusG binding on β’ clamp.

Biochemical analysis revealed that RfaH fails to accelerate pause-resistant, ‘fast’ RNAP variants that contain changes in regions located far from the RfaH-binding site (36). By contrast, pause-prone RNAPs were hypersensitive to RfaH. Furthermore, RfaH slowed down, rather than accelerated, transcription by the wild-type RNAP on a template lacking pause sites (36). This led us to propose that RfaH may act as an allosteric switch, which controls domain rearrangements that accompany transitions between elongating and paused states.

**Figure 7.**
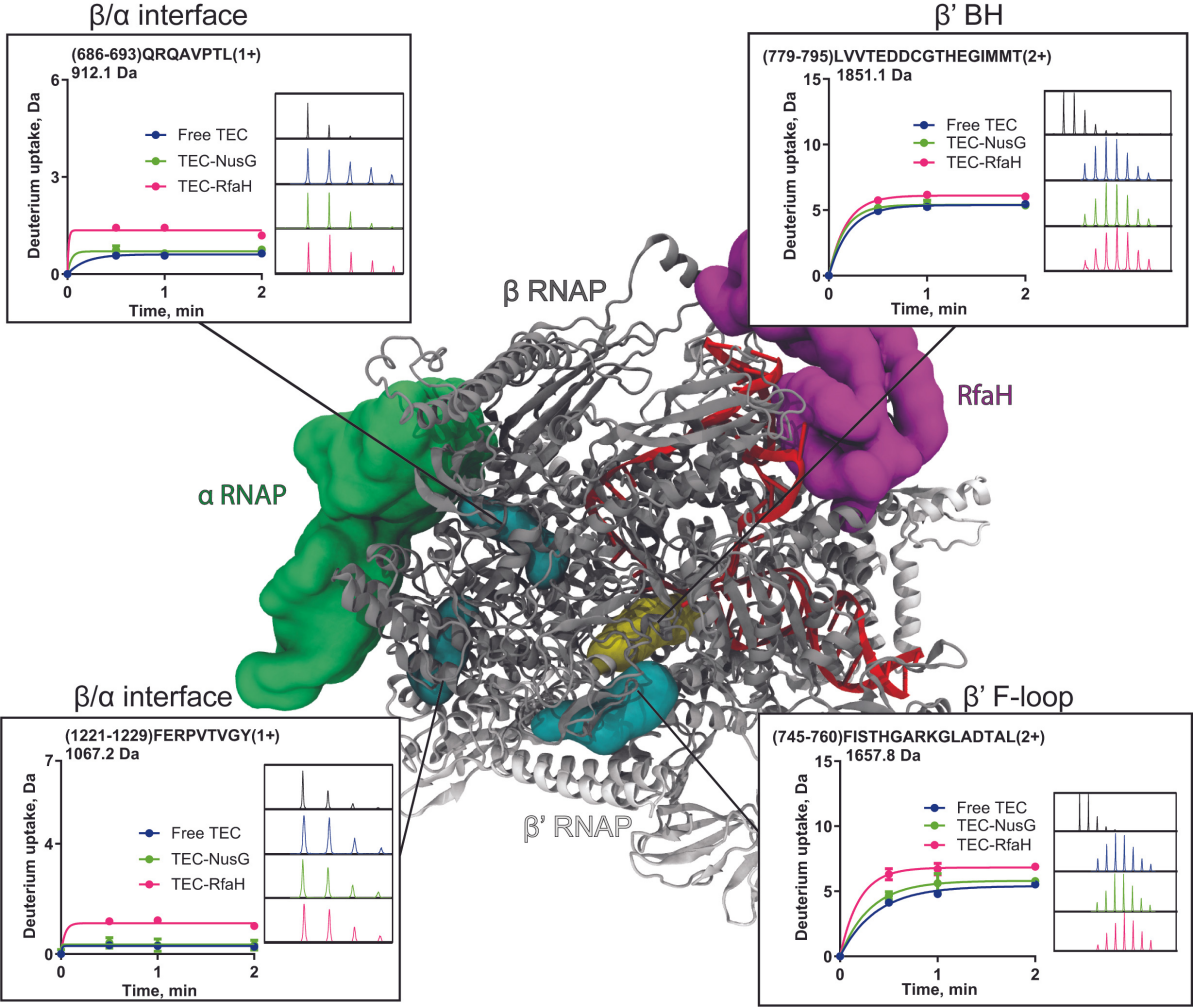
Changes in RNAP structural dynamics upon RfaH binding. The TEC structure (PDB 6c6u) is shown with subunits β (gray), β’ (white) and the DNA (red) in cartoon representation, while the α subunit (green) and the bound factor (purple) are displayed in surface representation. Peptides that exhibited significant changes in deuterium uptake upon RfaH binding and located on the interface between RNAP subunits are shown in surface representation and colored in cyan, while those interacting with nucleic acids are colored yellow. Each plot shows the deuterium uptake for factor-bound and free TEC over time, with the inset displaying the mass spectra of the nondeuterated sample (black) and after 2 min of exchange in deuterated buffer in the free (blue), NusG-bound (green) and RfaH-bound (pink) conditions.

In support of this model, we found that RfaH binding led to changes in deuterium uptake in RNAP regions that have been implicated in regulation of pausing and termination but are distant from the β’CH. RfaH – but not NusG – binding led to increased deuterium uptake in the β’ subunit peptide covering residues 779–795 in the catalytic bridge helix (BH), the most recognizable RNAP element (66) that interconnects the two pincers of RNAP (Figure 7, Supplementary Tables S6 and S7). Substitutions of S793 and Y795 residues make RNAP pause-prone and hypersensitive to RfaH (36). Similarly, binding of RfaH increased uptake by 1.4 deuterons in β’ F-loop (residues 745–760; Figure 7, Supplementary Table S7) located at the key β/β’ interface implicated in RNAP response to regulatory pauses (15). Disruption of this interface, composed of the β’ BH and F-loop and the β subunit D and Fork loops, by amino acid substitutions results in accelerated catalysis and reduced pausing (15), as well as insensitivity to RfaH (36). The quintessential fast RNAP with the β’ F773V substitution in the BH, isolated in a screen for mutants dependent on CBR antibiotics for survival (67), is characterized by error-prone catalysis, slow translocation and resistance to pause signals and RfaH (36). These phenotypes result from the loss of the β/β’ coupling that is essential for regulated transcription. Remarkably, binding of CBR restores coupling, and cell viability (15).

The β subunit–BH interactions also control the equilibrium between the active and paused states of the TEC via the BH anchor and switch 1 regions (68). We observed an increase of more than 4 deuterons in residues 1308–1321 (Supplementary Table S7) located in switch 1, where many termination-altering RNAP mutants map (69). However, we were unable to detect this region in the NusG-TEC complex.

Regarding regions out of central cleft, it was observed an increase in deuterium uptake at the β/α interface upon RfaH (β residues 686–693 and 1221–1229; Figure 7, Supplementary Table S5) and NusG binding (β residues 1305–1323; Supplementary Table S4).

We conclude that RfaH binding leads to changes in RNAP structural dynamics and inter-subunit interactions, manifested as allosteric control. By contrast, NusG binding produced only minor changes in RNAP.

## DISCUSSION

RNA chain elongation is accompanied by large conformational transitions in RNAP. Some of these changes occur in every nucleotide addition cycle, ∼50 times/sec; the β’ subunit trigger loop refolds into α helices and forms a triple-helical bundle with the bridge helix, positioning the substrate NTP for catalysis (14). Other changes demarcate rare regulatory events; movements of the RNAP clamp are thought to underpin TEC rearrangements into pause and termination states (65,68).

Diverse nucleic acid signals and accessory proteins are thought to influence these conformational transitions, but their effects have been challenging to investigate using traditional structural and biochemical approaches. Instead, RNAP dynamics has been initially inferred from the effects of antibiotics, which stabilize inactive intermediates; for example, streptolydigin traps an unfolded trigger loop (14) while myxopyronin locks the clamp movements by inducing refolding of the switch 2 region that serves as a hinge for clamp rotation (16). Recent cryo-electron microscopy imaging demonstrated that initiation (2), termination (12,13), and recycling factors (3,70) stabilize RNAP states with large conformational changes, particularly in the clamp. By contrast, NusG-like proteins that promote processive elongation would be expected to have more subtle effects, acting to minimize rather than to trigger dramatic changes in RNAP. The ubiquity of these proteins across all life argues that their maintenance function can be necessary for viability.

In this work, we used HDXMS to investigate changes in structural dynamics of *E. coli* NusG and RfaH that accompany their recruitment to TEC, as well as concomitant changes in RNAP. These factors represent housekeeping and highly specialized regulators, respectively, and display functional differences in their interactions with, and their effects on, the elongating RNAP (9,25,27,30,31).

Upon binding to *ops*-TEC, significant changes in deuterium uptake were observed for both factors. The residues in the last β strand and α-helix of the NTD consistently showed a decrease in deuterium uptake in both RfaH and NusG. These data are consistent with the structures of NusG- and RfaH-bound TEC, where these NTD regions interact with similar sites of the βGL and β’CH domains (20,27), and indicate that this region is stabilized upon binding to TEC. This effect is reciprocal, as both NusG and RfaH have been shown to stabilize the upstream edge of the transcription bubble (22,33) to inhibit RNAP backtracking.

Although both NusG and RfaH are positioned near the non-template DNA strand, only RfaH NTD establishes base-specific contacts with the *ops* hairpin (26,27). Substitutions for alanine in K10, R16, H20, T72 and R73 produced strong defects in DNA binding, whereas mutating residues Q13, R23, H65, T66 and T68 led to moderate defects (55). However, only K10, H20, R23 and R73 make direct contacts with *ops* (26). We observed a decrease in deuterium uptake for residues 72–78 in the RfaH-TEC complex (Figure 2B). Other residues close to this RfaH-RNAP binding site (Q13, R23, H65, T66, T67 and T68) showed increased uptake. Even though these residues do not interact directly with the *ops*, they may affect positioning of the DNA-interacting residues or interactions between RfaH and RNAP, showing a greater mobility upon TEC binding.

Other regions of RfaH NTD, such as the β-hairpin that makes up the interface with the CTD in the autoinhibited state (9), showed an increase in deuterium uptake upon TEC binding. For keeping RfaH autoinhibited, contacts between the β-hairpin and the interface are necessary (28,53,54). Disruption of the domain interface by TEC binding eliminates these contacts, which could partly explain the observed increase in deuterium exchange.

An increase in deuterium uptake upon TEC binding is also observed for the CTD of RfaH, but not NusG. While a sub-population of particles in cryoEM structures of RfaH-bound TEC suggests proximity between RNAP and β1-β2 of RfaH CTD (27), our results showed significant increase in its deuterium uptake (2.8 deuterons). Only residues 143–145, which comprise the ribosome binding region (29), show decreased deuteron uptake. In contrast, most of NusG CTD shows no significant changes excepting strands β3-β4 (residues 159–174), where a decrease in deuterium uptake is observed (Figure 4). In free NusG, the two domains move independently (71). Possibly, RNAP binding restricts the NusG CTD movements without establishing direct contacts. Consistent with our HDXMS observations, recent NMR studies revealed that the whole isolated RfaH CTD shows extensive and fast motions across all timescales that are absent in the isolated NusG CTD (59). Thus, our results indicate that the increased dynamics of RfaH CTD upon TEC binding is related to both its metamorphic behavior and to fast internal motions intrinsic to the β-folded RfaH CTD that is stabilized by NTD binding to the TEC.

All NusG homologs from archaea, bacteria, and eukaryotes make bridging contacts to the RNAP pincers proposed to restrict their mobility and prevent RNAP cleft opening prior to termination (7,35,65). Interactions with the β’CH are thought to make the principal contribution to binding affinity (9), whereas contacts with the βGL are critical for RfaH-mediated DNA restructuring and anti-pausing activity (7). We observed that binding of either RfaH or NusG reduced deuterium exchange in βGL. Structural and computational data suggest that the βGL is considerably more dynamic than the clamp domain (64), supporting a model in which the reduction of βGL mobility makes a key contribution to anti-pausing activity of NusG homologs (7). Neither protein appears to affect the clamp dynamics, and it is difficult to envision how a factor binding to the tip of a massive clamp domain would restrict its movements; indeed, ligands that restrain the clamp bind to the pivot point at its base (14–16). Why is the βGL dispensable for NusG activity (22,56)? We noted that the principal NusG effect on elongation is to inhibit backtracking (22), and contacts to the β’CH that position NusG at the edge of the DNA bubble would be sufficient for this activity (27). In addition, these contacts enable NusG to interact with Rho or the ribosome (in *E. coli*) (12,13,21,32,58), with specific DNA sequences (in *Bacillus subtilis*) (72) or with antitermination factors (73) (likely in many bacteria).

By contrast, RfaH is a potent anti-pausing factor able to counteract diverse pause signals and even weak intrinsic terminators (9,55). Anti-backtracking activity alone is insufficient to explain these effects and, unlike NusG, RfaH is also able to inhibit conformational changes in RNAP that accompany transitions to paused states (7,55). Decades of genetic and biochemical studies identified a set of fast, pause-resistant RNAP variants (36,67). Amino acid substitutions at the interface between β and β’ subunits, among which β’ F773V in the bridge helix has the most dramatic phenotype, abrogate tight coupling between the subunits and result in insensitivity to regulatory pauses; antibiotic CBR fills the gap and restores pausing (15,67). β’ F773V RNAP is completely resistant to RfaH (36), prompting a model in which RfaH induces allosteric changes that mimic the effect of this substitution (36,67). Remarkably, we found that RfaH binding leads to increased deuterium uptake at the β/β’ RNAP interface and in switch 1 and other regions which modulate clamp mobility upon RfaH binding. These changes in deuterium uptake suggest that RfaH binding to the TEC promotes both structural adaptations (i.e. changes on hydrogen bonding stability and solvent accessibility) and structural dynamics (i.e. changes on local internal motions and structural flexibility) in these distant regions to favor pause-free elongation.

In conclusion, our HDXMS results show differences in the local structural dynamics of RfaH and NusG, as well as in their effects on RNAP, upon interacting with TEC. These changes are discrete but localized in important regions related with factor binding, nucleic acid movement and RNAP oligomerization, including evidence of potential allosteric effects exerted by RfaH. We think that RfaH-induced changes in RNAP expand the extensive biochemical, structural, and microbiological studies of the role of RfaH in regulating gene expression and further promote our understanding of RfaH action during the expression of long virulence and conjugation operons in many Gram-negative bacterial pathogens.

Finally, it is important to highlight how HDXMS can be a useful tool for studying protein interactions and dynamics (74), even in large complexes or communicated over large distances. Recent HDXMS analyses revealed how RNAP interacts with PcrA helicase to maintain genome stability (75) or how sigma factors can recognize promoter DNA efficiently (76) and enabled the study of conformational changes in highly complex systems such as the *E. coli* Sec translocon (77). HDXMS has been also combined with X-ray crystallography, cryo-EM and molecular dynamics to understand cell signaling activation by ligand-receptor-effector interactions (78,79). These results and our present work demonstrate that HDXMS is a versatile addition to other structural, biochemical, and computational strategies to investigate the role of structural dynamics and allostery in protein folding, interaction, and function.

## DATA AVAILABILITY

Raw deuterium uptake data for RfaH, NusG and RNAP in the free and bound forms and maximum deuterium uptakes estimated from single exponential fitting can be found in the accompanying Supplementary Data.

## Supplementary Material

gkac453_Supplemental_File
