# Supplementary material for: Allosteric couplings upon binding of RfaH to transcription elongation complexes
Source: Nucleic Acids Res. 2022 Jun 7;50(11):6384–97. doi: 10.1093/nar/gkac453 (PMC9226497; doi:10.1093/nar/gkac453)
Supplement: gkac453_Supplemental_File [file gkac453_supplemental_file.pdf]

SUPPLEMENTARY INFORMATION

Allosteric couplings upon binding of RfaH to transcription elongation complexes

José Alejandro Molina[1,2], Pablo Galaz-Davison[1,2], Elizabeth A. Komives[3], Irina Artsimovitch[4*] and César A. Ramírez-Sarmiento[1,2*]

[1]Institute for Biological and Medical Engineering, Schools of Engineering, Medicine and Biological Sciences, Pontificia Universidad Católica de Chile, Santiago, Chile

[2]ANID – Millennium Science Initiative Program – Millennium Institute for Integrative Biology (iBio), Santiago, Chile

[3]Department of Chemistry and Biochemistry, University of California San Diego, La Jolla, California

[4]Department of Microbiology and The Center for RNA Biology, The Ohio State University, Columbus, Ohio

*Correspondence should be addressed to:

César A. Ramírez-Sarmiento, Institute for Biological and Medical Engineering, Schools of Engineering, Medicine and Biological Sciences, Pontificia Universidad Católica de Chile, Av. Vicuña Mackenna 4860, 7820436, Santiago, Chile; +56 2 2354 1110; cesar.ramirez@uc.cl

Irina Artsimovitch, Department of Microbiology and The Center for RNA Biology, The Ohio State University, 484 W. 12th Ave, Columbus, OH 43210, USA; +1 614 688 3561; artsimovitch.1@osu.edu

Nucleic Acid Sequences for Assembly of *ops*TEC

```
                        +1
5′- GGGCTGCGGTAGCGTGACGGCGAATACCC -3´   NT DNA

3′- CCCGACGCTCTCCATGTGCCGCTTATGGG -5′   T DNA

             CGAGAGGUA -3′                RNA
5′- GCAUUCAAAGC
```

ops: underlined sequence.

Determination of deuteron incorporation in overlapping peptides

In order to maximize sequence resolution through the analysis of overlapping peptides, the following considerations were made: (*i*) incorporation was calculated for the shortest available peptide and (*ii*) the deuteron incorporation of the overhanging region corresponds to the difference in incorporation between the two peptides for two overlapping peptides whose sequence differs only in one overhanging bit (i.e. ACE and ACEDF). For accuracy, the uncertainty (standard deviation, SD) of each individual peptide was considered and was propagated towards the difference peptide as the sum of their variances. If the resulting SD resulted in more than 20% of the differential incorporation along the time intervals, a longer peptide was used instead. For this analysis, only the incorporating amides were considered, therefore the maximum incorporation following equation:

$$N = L_{peptide} - n_{pro} - 1$$

$L_{peptide}$ corresponds to the length of the peptide and n_{pro} the number of proline residues contained in its sequence. The -1 arises from the fast exchange that takes place at the N-terminal of the protein or peptic peptides. However, for most overlapping peptides, the fast exchange of the N-terminal is already considered, thus their maximum incorporation was not corrected again for fast exchange.

Determination of peptides with significant differences between bound and free states

ANOVA analyses and t-tests with a p-value cutoff of 0.05 implemented in the program DECA, were used to determine the significance of differences in HDX between bound and free state. These analyses were calculated using the software DECA v1.14 available in https://github.com/komiveslab/DECA. Supplementary Figure S3 shows the confidence intervals for peptides shown in Figures 2, 4, 6 and 7 in the main text, which have statistically significant differences according to ANOVA analysis to the peptides. In general terms, HDX differences greater than ~1 deuterons are significant. However, according to ANOVA analyses, smaller differences of 0.4 deuterons are statistically significant, as in the case of NusG and RNAP allostery cases (Supplementary Tables S2, S5 and S7).

HDXMS data were fitted to a single exponential to obtain the maximum deuteron incorporation per peptide (Tables S1-S7). The rates derived from these data fits are not reported in these tables, as they are not very informative given the exchange reaction times employed in our work. Nevertheless, they can be calculated from the raw data in Supplementary Tables S8-S14.

Supplementary Table S1 Deuteron incorporation of RfaH

Position	Sequence	TEC-RfaH		Free RfaH		Δmass (TEC-free)	Δ % Incorp (TEC-free)
		Δmass. AMU	%Incorp.	Δmass. AMU	%Incorp.		
1-7	MQSWYLL	1.1 ± 0.3	18.3	1.2 ± 0.1	20.0	-0.1	-1.7
8-19	YCKRGQLQRAQE	8.0 ± 0.5	72.7	5.7 ± 0.1	51.8	2.3	20.9
20-29	HLERQAVNCL	4.3 ± 0.2	47.8	3.9 ± 0.2	43.3	0.4	4.4
22-29[a]	ERQAVNCL	4.3 ± 0.2	61.4	2.9 ± 0.1	41.4	1.4	20.0
22-34[a]	ERQAVNCLAPMIT	7.0 ± 0.1	63.6	4.9 ± 0.2	44.5	2.1	19.1
30-34[a]	APMIT	2.7 ± 0.2	67.5	2.0 ± 0.1	50.0	0.7	17.5
35-55	LEKIVRGKRTAVSEPLFPNYL	13.0 ± 0.8	72.2	7.5 ± 0.4	41.7	5.5	30.6
57-71[b]	VEFDPEVIHTTTINA	9.6 ± 0.3	73.8	6.6 ± 0.4	50.8	3	23.1
57-78[b]	VEFDPEVIHTTTINATRGVSHF	13.6 ± 0.9	68.0	11.4 ± 0.4	57.0	2.2	11.0
72-78[b]	TRGVSHF	3.9 ± 0.1	55.7	4.8 ± 0.2	68.6	-0.9	-12.9
79-95	VRFGASPAIVPSAVIHQ	5.8 ± 0.7	41.4	6.8 ± 0.1	48.6	-1	-7.1
96-107[c]	LSVYKPKDIVDP(ENL)	11.3 ± 0.4	94.2	6.1 ± 0.3	50.8	5.2	43.3
104-107[c]	IVDP(ENL)	4.2 ± 0.2	84.0	2.3 ± 0.2	46.0	1.9	38.0
96-103[c]	LSVYKPKD	7.0 ± 0.2	100.0	3.8 ± 0.1	54.3	3.2	45.7
108-120	(YFQG)ATPYPGDKVIITE	13.2 ± 0.8	94.3	7.0 ± 0.5	50.0	6.2	44.3
121-129	GAFEGFQAI	8.0 ± 0.1	100.0	4.4 ± 0.2	55.0	3.6	45.0
130-141	FTEPDGEARSML	6 ± 1	60.0	3.7 ± 0.7	37.0	2.3	23.0
142-159[d]	LLNLINKEIKHSVKNTEF	12.6 ± 0.7	70.6	7.1 ± 0.4	41.8	4.9	28.8
146-159[d]	INKEIKHSVKNTEF	9.7 ± 0.6	74.6	5.3 ± 0.3	40.8	4.4	33.8
142-145[d]	LLNL	3.1 ± 0.2	77.5	1.8 ± 0.1	45.0	1.3	32.5

Expressed as average ± std. error of fit
(ENL) and (YFQG) correspond to the TEV cleaving sequence and was not considered in the sequence numbering. a-d Deuteron incorporations in red were estimated from the difference between two overlapping peptides.

Supplementary Table S2 Deuteron incorporation of NusG

Position	Sequence	TEC-NusG		Free NusG		Δmass (TEC-free)	Δ % Incorp (TEC-free)
		Δmass. AMU	%Incorp.	Δmass. AMU	%Incorp.		
2-14	SEAPKKRWYVVQA	5.0 ± 0.7	45.5	6.1 ± 0.7	55.5	-1.1	-10.0
14-28	AFSGFEGRVATSL	9.3 ± 0.5	77.5	9.7 ± 0.2	80.8	-0.4	-3.3
29-38	HIKLHNMEDL	4.5 ± 0.4	50.0	4.6 ± 0.6	51.1	-0.1	-1.1
41-48	EVMVPTEE	4.6 ± 0.1	76.7	4.9 ± 0.3	81.7	-0.3	-5.0
49-70	VVEIRGGQRRKSERKFFPGYVL	12.0 ± 0.3	60.0	11 ± 0.6	55.0	1.0	5.0
71-80[a]	VQMVMNDASW	4.2 ± 0.4	46.7	4.6 ± 0.6	51.1	-0.4	-4.4
74-80[a]	VMNDASW	2.8 ± 0.3	46.7	3.2 ± 0.4	53.3	-0.4	-6.7
71-73[a]	VQM	1.4 ± 0.1	70.0	1.5 ± 0.1	75.0	-0.1	-5.0
81-109[b]	HLVRSVPRVMGFIGGTSDRPAPISDKEVD	19.9 ± 0.6	79.6	20.3 ± 0.4	81.2	-0.4	-1.6
92-109[b]	FIGGTSDRPAPISDKEVD	11.4 ± 0.3	76.0	12.7 ± 0.2	84.7	-1.3	-8.7
81-91[b]	HLVRSVPRVMG	8.5 ± 0.3	94.4	7.6 ± 0.3	84.4	0.9	10.0
110-133	AIMNRLQQVGDKPRPKTLFEPGEM	18.9 ± 0.3	94.5	19.2 ± 0.7	96.0	-0.3	-1.5
134-144[c]	VRVNDGPFADF	6.4 ± 0.5	71.1	6.5 ± 0.6	72.2	-0.1	-1.1
134-150[c]	VRVNDGPFADFNGVVEE	9.3 ± 0.9	60.0	9.5 ± 0.9	66.7	-0.2	-6.7
145-150[c]	NGVVEE	2.9 ± 0.6	48.3	3.2 ± 0.7	53.3	-0.3	-5.0
151-158	VDYEKSRL	5.3 ± 0.4	75.7	5.4 ± 0.5	77.1	-0.1	-1.4
159-165[d]	KVSVSIF	5.1 ± 0.2	85.0	5.2 ± 0.4	86.7	-0.1	-1.7
159-174[d]	KVSVSIFGRATPVELD	10.6 ± 0.1	75.7	12.4 ± 0.9	88.6	-1.8	-12.9
166-174[d]	GRATPVELD	5.7 ± 0.4	71.3	7.1 ± 0.5	88.8	-1.4	-17.5
174-181	DFSQVEKA	4.6 ± 0.5	65.7	5.1 ± 0.7	72.9	-0.5	-7.1

Expressed as average ± std. error of fit
a-d: Deuteron incorporations in red were estimated from the difference between two overlapping peptides.

Supplementary Table S3 Deuteron incorporation of isolated CTD of RfaH

Position	Sequence	Δmass. AMU	%Incorp.
117-123	IITEGAF	3.9 ± 0.1	65.0
124-142[a]	EGFQAIFTEPDGEARSMLL	12.6 ± 0.2	74.1
130-142[a]	FTEPDGEARSMLL	6.6 ± 0.1	60.0
124-129[a]	EGFQAI	5.9 ± 0.1	98.3
143-158[b]	LNLINKEIKHSVKNTE	9.8 ± 0.3	61.3
146-158[b]	INKEIKHSVKNTE	7.1 ± 0.2	59.2
143-145[b]	LNL	2.6 ± 0.1	86.7
146-159	INKEIKHSVKNTEF	7.1 ± 0.2	54.6

Expressed as average ± std. error of fit
a-b: Deuteron incorporations in red were estimated from the difference between two overlapping peptides.

Supplementary Table S4 Deuteron incorporation of RNAP β subunit upon NusG binding

Position	Sequence	TEC-NusG		Free TEC		Δmass (Bound-free)	Δ % Incorp (Bound-free)
		Δmass. AMU	%Incorp.	Δmass. AMU	%Incorp.		
53-68	FRSVFPIQSYSGNSEL	6.3 ± 0.3	45.0	6.7 ± 0.2	47.9	-0.4	-2.9
69-80	QYVSYRLGEPVF	6.2 ± 0.2	62.0	6.1 ± 0.2	61.0	0.1	1.0
81-102	DVQECQIRGVTYSAPLRVKLRL	7.1 ± 0.3	35.5	7.4 ± 0.2	37.0	-0.3	-1.5
103-121	VIYEREAPEGTVKDIKEQE	14.4 ± 0.1	84.7	14.8 ± 0.3	87.1	-0.4	-2.4
120-136	QEVYMGEIPLMTDNGTF	3.8 ± 0.1	25.3	4.1 ± 0.1	27.3	-0.3	-2.0
137-149	VINGTERVIVSQL	1.9 ± 0.1	15.8	2.0 ± 0.1	16.7	-0.1	-0.8
157-183	FDSDKGKTHSSGKVLYNARIIPYRGSW	7.6 ± 0.1	30.4	7.2 ± 0.2	28.8	0.4	1.6
187-195	EFDPKDNLF	1.9 ± 0.1	27.1	1.6 ± 0.1	22.9	0.3	4.3
211-223	RALNYTTEQILDL	3.1 ± 0.1	25.8	3.0 ± 0.1	25.0	0.1	0.8
224-230	FFEKVIF	3.3 ± 0.1	55.0	3.2 ± 0.1	53.3	0.1	1.7
231-239	EIRDNKLQM	4.2 ± 0.2	52.5	4.3 ± 0.1	53.8	-0.1	-1.3
240-253	ELVPERLRGETASF	7.4 ± 0.7	61.7	7.3 ± 0.4	60.8	0.1	0.8
322-336	LAKLSQSGHKRIETL	4.3 ± 0.2	30.7	4.1 ± 0.2	29.3	0.2	1.4
337-349	FTNDLDHGPYISE	4.9 ± 0.1	44.5	5.1 ± 0.1	46.4	-0.2	-1.8
351-363	LRVDPTNDRLSAL	4.7 ± 0.1	42.7	5.1 ± 0.2	46.4	-0.4	-3.6
364-384	VEIYRMMRPGEPPTREAAESL	8.3 ± 0.5	48.8	9.6 ± 0.2	56.5	-1.3	-7.6
389-399	FFSEDRYDLSA	3.4 ± 0.1	34.0	3.5 ± 0.2	35.0	-0.1	-1.0
400-412	VGRMKFNRSLLRE	3.8 ± 0.5	31.7	3.9 ± 0.3	32.5	-0.1	-0.8
413-428	EIEGSGILSKDDIIDV	5.5 ± 0.1	36.7	5.7 ± 0.1	38.0	-0.2	-1.3
460-468	AENQFRVGL	5.1 ± 0.2	63.8	4.0 ± 0.1	50.0	1.1	13.8
485-500	DTLMPQDMINAKPISA	10.2 ± 0.6	78.5	10.5 ± 0.6	80.8	-0.3	-2.3
501-514	AVKEFFGSSQLSQF	6.3 ± 0.3	48.5	6.6 ± 0.4	50.8	-0.3	-2.3
546-574	EVRDVHPTHYGRVCPIETPEGPNIGLINS	4.0 ± 0.1	16.7	3.5 ± 0.1	14.6	0.5	2.1
587-608	LETPYRKVTDGVVTDEIHYLSA	7.2 ± 0.1	36.0	7.3 ± 0.2	36.5	-0.1	-0.5
624-632	DEEGHFVED	5.0 ± 0.3	62.5	5.0 ± 0.3	62.5	0	0.0
645-652	FSRDQVDY	2.0 ± 0.1	28.6	2.1 ± 0.1	30.0	-0.1	-1.4
665-681	ASLIPFLEHDDANRALM	2.6 ± 0.3	17.3	2.5 ± 0.1	16.7	0.1	0.7
686-693	QRQAVPTL	0.7 ± 0.1	11.7	0.6 ± 0.1	10.0	0.1	1.7
694-707	RADKPLVGTGMERA	4.4 ± 0.4	36.7	4.4 ± 0.2	36.7	0	0.0
708-716	VAVDSGVTA	2.8 ± 0.1	35.0	2.7 ± 0.3	33.8	0.1	1.3
750-772	IYNLTKYTRSNQNTCINQMPCVS	4.6 ± 0.2	21.9	4.4 ± 0.3	21.0	0.2	1.0
773-783	LGEPVERGDVL	2.9 ± 0.1	22.2	2.1 ± 0.2	23.3	-0.1	-1.1
800-811	MRVAFMPWNGYN	2.5 ± 0.2	25.0	2.5 ± 0.2	25.0	0	0.0
964-971	LQILEAGL	2.6 ± 0.2	37.1	2.8 ± 0.3	40.0	-0.2	-2.9
972-979	FSRIRAVL	1.2 ± 0.1	17.1	1.2 ± 0.1	17.1	0	0.0
998-1014	LELGLTDEEKQNQLEQL	12.4 ± 0.5	77.5	12.6 ± 0.3	78.8	-0.2	-1.3

1048-1054	KIVKVYL	0.1 ± 0.1	1.7	0.1 ± 0.1	1.7	0	0.0
1096-1107	IVLNPLGVPSRM	5.8 ± 0.1	64.4	6.0 ± 0.1	66.7	-0.2	-2.2
1108-1115	NIGQILET	0.6 ± 0.2	8.6	0.6 ± 0.1	8.6	0	0.0
1131-1143	MLKQQQEVAKLRE	9.4 ± 0.4	78.3	9.4 ± 0.2	78.3	0	0.0
1144-1153	FIQRAYDLGA	2.5 ± 0.1	27.8	2.5 ± 0.1	18.9	0.8	8.9
1173-1184	AENLRKGMPIAT	2.0 ± 0.1	20.0	1.9 ± 0.1	19.0	0.1	1.0
1185-1194	PVFDGAKEAE	3.8 ± 0.2	47.5	3.8 ± 0.3	47.5	0	0.0
1198-1212	LLKLGDLPTSGQIRL	5.1 ± 0.1	39.2	5.3 ± 0.1	40.8	-0.2	-1.5
1213-1220	YDGRTGEQ	2.1 ± 0.1	30.0	1.9 ± 0.1	27.1	0.2	2.9
1221-1229	FERPVTVGY	0.3 ± 0.1	4.3	0.3 ± 0.1	4.3	0	0.0
1291-1304	LTVKSDDVNGRTKM	6.3 ± 0.1	48.5	6.2 ± 0.5	47.7	0.1	0.8
1305-1323	YKNIVDGNHQMEPGMPESF	4.7 ± 0.3	29.4	5.7 ± 0.1	35.6	-1	-6.3
1327-1336	LKEIRSLGIN	0.4 ± 0.1	4.4	0.3 ± 0.1	3.3	0.1	1.1

Expressed as average ± std. error of fit

Supplementary Table S5 Deuteron incorporation of RNAP β subunit upon RfaH binding.

Position	Sequence	TEC-RfaH		Free TEC		Δmass (Bound-free)	Δ % Incorp (Bound-free)
		Δmass. AMU	%Incorp.	Δmass. AMU	%Incorp.		
39-52	IEQDPEGQYGLEAA	6.2 ± 0.5	51.7	3.8 ± 0.2	31.7	2.4	20.0
53-68	FRSVFPIQSYSGNSEL	7 ± 0.3	50.0	6.7 ± 0.2	47.9	0.3	2.1
69-80	QYVSYRLGEPVF	6.8 ± 0.5	68.0	6.1 ± 0.2	61.0	0.7	7.0
103-121	VIYEREAPEGTVKDIKEQE	15.0 ± 0.7	88.2	14.8 ± 0.3	87.1	0.2	1.2
137-149	VINGTERVIVSQL	2.8 ± 0.2	23.3	2.0 ± 0.1	1.4	0.8	21.9
150-156	HRSPGVF	1.0 ± 0.1	20.0	1.1 ± 0.1	22.0	-0.1	-2.0
175-183	RIIPYRGSW	1.9 ± 0.1	27.1	1.6 ± 0.1	22.9	0.3	4.3
187-194	EFDPKDNL	1.7 ± 0.1	28.3	1.5 ± 0.1	*25.0*	0.2	3.3
211-223	RALNYTTEQILDL	3.1 ± 0.2	25.8	3.0 ± 0.1	25.0	0.1	0.8
224-230	FFEKVIF	3.4 ± 0.2	56.7	3.2 ± 0.1	53.3	0.2	3.3
231-239	EIRDNKLQM	4.4 ± 0.3	55.0	4.3 ± 0.1	53.8	0.1	1.3
240-253	ELVPERLRGETASF	7.5 ± 0.5	62.5	7.3 ± 0.4	60.8	0.2	1.7
285-291	IEVPVEY	2.6 ± 0.2	52.0	2.6 ± 0.1	52.0	0	0.0
337-349	FTNDLDHGPYISE	5.5 ± 0.1	50.0	5.1 ± 0.1	46.4	0.4	3.6
351-360	TLRVDPTNDRLSA	3.9 ± 0.2	48.8	4.2 ± 0.1	52.5	-0.3	-3.8
364-384	VEIYRMMRPGEPPTREAAESL	7.3 ± 0.3	42.9	9.6 ± 0.2	56.5	-2.3	-13.5
390-399	FSEDRYDLSA	3.9 ± 0.1	43.3	3.6 ± 0.1	40.0	0.3	3.3
400-410	VGRMKFNRSLL	3.3 ± 0.2	27.5	2.8 ± 0.3	23.3	0.5	4.2
460-468	AENQFRVGL	3.1 ± 0.1	38.8	4.0 ± 0.1	50.0	-0.9	-11.3
501-514	AVKEFFGSSQLSQF	6.5 ± 0.3	50.0	6.6 ± 0.4	50.8	-0.1	-0.8
546-574	EVRDVHPTHYGRVCPIETPEGPNIGLINS	4.3 ± 0.1	17.9	3.5 ± 0.1	14.6	0.8	3.3
587-608	LETPYRKVTDGVVTDEIHYLSA	7.1 ± 0.1	35.5	7.3 ± 0.2	36.5	-0.2	-1.0
624-632	DEEGHFVED	5.3 ± 0.2	66.3	5.0 ± 0.3	62.5	0.3	3.8
645-653	FSRDQVDYM	2.3 ± 0.1	28.8	2.1 ± 0.1	26.3	0.2	2.5
686-693	QRQAVPTL	1.4 ± 0.2	23.3	0.6 ± 0.1	10.0	0.8	13.3
694-709	RADKPLVGTGMERAVA	4.9 ± 0.2	35.0	4.4 ± 0.2	31.4	0.5	3.6
708-716	VAVDSGVTA	3.0 ± 0.1	37.5	2.7 ± 0.3	33.8	0.3	3.8
717-729	VAKRGGVVQYVDA	2.9 ± 0.1	24.2	3.2 ± 0.3	26.7	-0.3	-2.5
750-772	IYNLTKYTRSNQNTCINQMPCVS	5.3 ± 0.2	25.2	4.4 ± 0.3	21.0	0.9	4.3
773-783	LGEPVERGDVL	2.0 ± 0.1	22.2	2.1 ± 0.2	23.3	-0.1	-1.1
876-883	EVTGGDIL	3.4 ± 0.1	48.6	3.2 ± 0.1	45.7	0.2	2.9
951-964	MQLKQAKKDLSEEL	8.4 ± 0.6	64.6	7.9 ± 0.5	60.8	0.5	3.8
965-971	QILEAGL	2.5 ± 0.3	41.7	2.4 ± 0.2	40.0	0.1	1.7
972-979	FSRIRAVL	1.3 ± 0.1	18.6	1.2 ± 0.1	17.1	0.1	1.4
980-997	VAGGVEAEKLDKLPRDRW	14.5 ± 0.8	90.6	14.0 ± 0.1	87.5	0.5	3.1

1048-1054	KIVKVYL	0.1 ± 0.1	1.7	0.1 ± 0.1	1.7	0	0.0
1096-1115	IVLNPLGVPSRMNIGQILET	7.2 ± 0.7	42.4	6.2 ± 0.1	36.5	1	5.9
1144-1153	FIQRAYDLGA	2.8 ± 0.1	31.1	2.5 ± 0.1	18.9	1.1	12.2
1173-1184	AENLRKGMPIAT	2.0 ± 0.2	20.0	1.9 ± 0.1	19.0	0.1	1.0
1185-1194	PVFDGAKEAE	4.4 ± 0.1	55.0	3.8 ± 0.3	47.5	0.6	7.5
1198-1212	LLKLGDLPTSGQIRL	5.9 ± 0.3	45.4	5.3 ± 0.1	40.8	0.6	4.6
1221-1229	FERPVTVGY	1.1 ± 0.2	15.7	0.3 ± 0.1	4.3	0.8	11.4
1254-1273	VTQQPLGGKAQFGGQRFGEM	9.4 ± 0.1	52.2	9.1 ± 0.3	50.6	0.3	1.7
1327-1336	LKEIRSLGIN	1.6 ± 0.7	17.8	0.3 ± 0.1	3.3	1.3	14.4

Expressed as average ± std. error of fit

Supplementary Table S6 Deuteron incorporation of RNAP β' subunit upon NusG binding.

Position	Sequence	TEC-NusG		Free TEC		Δmass (Bound-free)	Δ % Incorp (Bound-free)
		Δmass. AMU	%Incorp.	Δmass. AMU	%Incorp.		
89-99	LKSLPSRIGLL	3.9 ± 0.1	43.3	3.9 ± 0.2	43.3	0	0.0
100-112	LDMPLRDIERVLY	2.8 ± 0.1	25.5	2.8 ± 0.1	25.5	0	0.0
127-136	ERQQILTEEQ	2.0 ± 0.1	22.2	1.9 ± 0.1	21.1	0.1	1.1
141-148	LEEFGDEF	3.0 ± 0.3	42.9	3.1 ± 0.3	44.3	-0.1	-1.4
212-232	TVLPVLPPDLRPLVPLDGGRF	8.1 ± 0.1	54.0	9.7 ± 0.1	64.7	-1.6	-10.7
260-273	PDIIVRNEKRMLQE	2.5 ± 0.1	20.8	6.3 ± 0.2	52.5	-3.8	-31.7
326-335	VITVGPYLRL	1.5 ± 0.1	18.8	1.5 ± 0.1	18.8	0	0.0
347-359	ELFKPFIYGKLEL	3.6 ± 0.1	32.7	3.5 ± 0.2	31.8	0.1	0.9
379-386	VVWDILDE	1.8 ± 0.2	25.7	1.5 ± 0.1	21.4	0.3	4.3
395-413	LNRAPTLHRLGIQAFEPVL	3.2 ± 0.3	20.0	2.9 ± 0.2	18.1	0.3	1.9
414-426	IEGKAIQLHPLVC	2.5 ± 0.1	22.7	2.4 ± 0.1	21.8	0.1	0.9
439-446	AVHVPLTL	0.4 ± 0.2	6.7	0.9 ± 0.1	15.0	-0.5	-8.3
581-592	YRILGLKPTVIF	2.4 ± 0.1	24.0	2.3 ± 0.1	23.0	0.1	1.0
616-628	MVIPEKKHEIISE	3.8 ± 0.3	34.5	3.8 ± 0.3	34.5	0	0.0
745-760	FISTHGARKGLADTAL	5.8 ± 0.1	38.7	5.4 ± 0.4	36.0	0.4	2.7
796-818	PVIEGGDVKEPLRDRVLGRVTAE	11.2 ± 0.1	56.0	11.3 ± 0.2	56.5	-0.1	-0.5
828-836	ILVPRNTLL	2.1 ± 0.2	30.0	2.0 ± 0.1	28.6	0.1	1.4
875-891	LARGHIINKGEAIGVIA	3.4 ± 0.1	21.3	3.2 ± 0.1	20.0	0.2	1.3
903-921	TMRTFHIGGAASRAAAESS	14.6 ± 0.2	81.1	15.3 ± 0.5	85.0	-0.7	-3.9
946-956	VITSRNTELKL	3.7 ± 0.1	37.0	3.8 ± 0.3	38.0	-0.1	-1.0
960-975	FGRTKESYKVPYGAVL	4.5 ± 0.1	32.1	4.3 ± 0.1	30.7	0.2	1.4
976-1002	AKGDGEQVAGGETVANWDPHTMPVITE	13.5 ± 0.4	56.3	13.8 ± 0.5	57.5	-0.3	-1.3
1087-1093	ISSGDTL	2.1 ± 0.1	35.0	2.3 ± 0.1	38.3	-0.2	-3.3
1094-1116	ARIPQESGGTKDITGGLPRVADL	11.8 ± 0.2	59.0	12.1 ± 0.1	60.5	-0.3	-1.5
1117-1128	FEARRPKEPAIL	4.7 ± 0.1	52.2	4.8 ± 0.1	53.3	-0.1	-1.1
1129-1137	AEISGIVSF	1.6 ± 0.1	20.0	1.7 ± 0.3	21.3	-0.1	-1.3
1138-1161	GKETKGKRRLVITPVDGSDPYEEM	7.4 ± 0.3	35.2	5.1 ± 0.3	24.3	2.3	11.0
1209-1215	VQDVYRL	1.8 ± 0.1	30.0	1.5 ± 0.1	25.0	0.3	5.0
1298-1306	QETTRVLTE	2.7 ± 0.1	33.8	2.9 ± 0.1	43.3	-0.2	-2.5

Expressed as average ± std. error of fit

Supplementary Table S7 Deuteron incorporation of RNAP β' subunit upon RfaH binding.

Position	Sequence	TEC-RfaH		Free TEC		Δmass (Bound-free)	Δ % Incorp (Bound-free)
		Δmass. AMU	%Incorp.	Δmass. AMU	%Incorp.		
90-100	KSLPSRIGLLL	4.2 ± 0.3	46.7	3.9 ± 0.2	43.3	0.3	3.3
100-108	LDMPLRDIE	2.7 ± 0.1	38.6	2.7 ± 0.1	38.6	0	0.0
127-136	ERQQILTEEQ	2.5 ± 0.1	27.8	1.9 ± 0.1	21.1	0.6	6.7
141-148	LEEFGDEF	3.3 ± 0.4	47.1	3.1 ± 0.3	44.3	0.2	2.9
199-208	FVQSGNKPEW	4.3 ± 0.3	53.8	4.2 ± 0.2	52.5	0.1	1.3
215-232	PVLPPDLRPLVPLDGGRF	8.9 ± 0.3	74.2	9.5 ± 0.1	79.2	-0.6	-5.0
233-240	ATSDLNDL	3.6 ± 0.2	50.0	3.6 ± 0.1	51.4	-0.1	-1.4
260-273	PDIIVRNEKRMLQE	3.0 ± 0.4	25.0	6.3 ± 0.2	52.5	-3.3	-27.5
326-335	VITVGPYLRL	1.7 ± 0.2	21.3	1.5 ± 0.1	18.8	0.2	2.5
347-359	ELFKPFIYGKLEL	3.9 ± 0.1	35.5	3.5 ± 0.2	31.8	0.4	3.6
379-386	VVWDILDE	1.7 ± 0.1	24.3	1.5 ± 0.1	21.4	0.2	2.9
387-394	VIREHPVL	1.3 ± 0.1	21.7	1.3 ± 0.1	21.7	0	0.0
414-426	IEGKAIQLHPLVC	2.7 ± 0.4	24.5	2.4 ± 0.1	21.8	0.3	2.7
439-446	AVHVPLTL	0.7 ± 0.4	11.7	0.9 ± 0.1	15.0	-0.2	-3.3
536-551	VAKTSLKDTTVGRAIL	3.0 ± 0.1	20.0	2.4 ± 0.1	16.0	0.6	4.0
581-592	YRILGLKPTVIF	2.3 ± 0.1	23.0	2.3 ± 0.1	23.0	0	0.0
616-628	MVIPEKKHEIISE	4.8 ± 0.1	43.6	3.8 ± 0.3	34.5	1	9.1
674-691	QTETVINRDGQEEKQVSF	16.1 ± 0.9	94.7	15.4 ± 0.3	90.6	0.7	4.1
716-729	RGLMAKPDGSIIET	8.5 ± 0.6	70.8	7.9 ± 0.3	65.8	0.6	5.0
736-744	REGLNVLQY	4.5 ± 0.3	56.3	4.4 ± 0.3	55.0	0.1	1.2
745-760	FISTHGARKGLADTAL	6.8 ± 0.1	45.3	5.4 ± 0.4	36.0	1.4	9.3
779-795	LVVTEDDCGTHEGIMMT	4.2 ± 0.1	26.3	3.7 ± 0.1	23.1	0.5	3.1
796-802	PVIEGGD	4.4 ± 0.2	88.0	4.8 ± 0.1	96.0	-0.4	-8.0
828-836	ILVPRNTLL	2.3 ± 0.3	32.9	2.0 ± 0.1	28.6	0.3	4.3
851-864	AVKVRSVVSCDTDF	3.9 ± 0.2	30.0	3.7 ± 0.1	28.5	0.2	1.5
875-891	LARGHIINKGEAIGVIA	3.8 ± 0.4	23.8	3.2 ± 0.1	20.0	0.6	3.8
946-956	VITSRNTELKL	4.2 ± 0.1	42.0	3.8 ± 0.3	38.0	0.4	4.0
960-975	FGRTKESYKVPYGAVL	5.2 ± 0.1	37.1	4.3 ± 0.1	30.7	0.9	6.4
1013-1028	IDGQTITRQTDELTGL	11.2 ± 0.3	74.7	11.4 ± 0.1	76.0	-0.2	-1.3
1060-1070	VLIPGTDMPAQ	4.5 ± 0.4	56.3	4.2 ± 0.2	52.5	0.3	3.8
1071-1077	YFLPGKA	1.6 ± 0.1	32.0	1,6 ± 0.1	32.0	0	0.0
1087-1093	ISSGDTL	2.2 ± 0.1	36.7	2.3 ± 0.1	38.3	-0.1	-1.7
1117-1128	FEARRPKEPAIL	5.0 ± 0.1	55.6	4.8 ± 0.1	53.3	0.2	2.2
1129-1137	AEISGIVSF	1.8 ± 0.1	22.5	1.7 ± 0.3	21.3	0.1	1.3
1228-1245	IVRQMLRKATIVNAGSSD	5.4 ± 0.1	31.8	4.9 ± 0.1	28.8	0.5	2.9

1280-1286	GITKASL	1.3 ± 0.4	21.7	0.8 ± 0.1	13.3	0.5	8.3
1298-1306	QETTRVLTE	3.3 ± 0.2	41.3	2.9 ± 0.1	36.3	0.4	5.0
1308-1321	AVAGKRDELRGLKE	7.3 ± 0.5	56.2	2.3 ± 0.1	17.7	5	38.5

Expressed as average ± std. error of fit

Supplementary Table S8. Deuteron incorporation for RfaH under different reaction times.

Position	Sequence	Mass, Da	tr(0), min	Deuteron incorporation, AMU ± SD				State
				0 min	0.5 min	1.0 min	2.0 min	
1-7	MQSWYLL	940.5	4.83	0	1.23 ± 0.02	1.26 ± 0.03	1.13 ± 0.01	Free
			4.99	0	0.83 ± 0.09	0.83 ± 0.03	1.18 ± 0.01	Bound
8-19	YCKRGQLQRAQE	1479.7	2.32	0	5.5 ± 0.1	5.8 ± 0.1	5.7 ± 0.1	Free
			2.14	0	7.3 ± 0.2	7.7 ± 0.1	8.3 ± 0.1	Bound
20-29	HLERQAVNCL	1182.6	3.40	0	3.76 ± 0.07	3.93 ± 0.09	3.81 ± 0.06	Free
			3.26	0	3.9 ± 0.2	4.1 ± 0.2	4.4 ± 0.1	Bound
22-29	ERQAVNCL	932.5	3.51	0	2.82 ± 0.06	2.93 ± 0.06	2.86 ± 0.04	Free
			3.41	0	3.94 ± 0.07	4.16 ± 0.04	4.42 ± 0.06	Bound
22-34	ERQAVNCLAPMIT	1445.7	4.02	0	4.8 ± 0.1	5.0 ± 0.1	4.8 ± 0.2	Free
			4.03	0	6.2 ± 0.1	6.7 ± 0.1	7.2 ± 0.1	Bound
35-55	LEKIVRGKRTAVSEPLFPNYL	2430.4	3.96	0	7.6 ± 0.2	7.7 ± 0.1	7.3 ± 0.1	Free
			3.93	0	12.4 ± 0.3	12.5 ± 0.2	13.4 ± 0.2	Bound
57-71	VEFDPEVIHTTTINA	1685.8	3.88	0	6.2 ± 0.2	6.4 ± 0.1	6.4 ± 0.1	Free
			4.15	0	9.3 ± 0.3	9.4 ± 0.1	9.73 ± 0.07	Bound
57-78	VEFDPEVIHTTTINATRGVSHF	2470.2	3.94	0	11 ± 0.2	11.4 ± 0.1	11.1 ± 0.1	Free
			3.99	0	12.8 ± 0.9	13 ± 0.3	14 ± 0.2	Bound
79-95	VRFGASPAIVPSAVIHQ	1749.0	3.67	0	6.6 ± 0.1	6.8 ± 0.2	6.7 ± 0.1	Free
			3.69	0	3.46 ± 0.01	4.35 ± 0.02	5.74 ± 0.03	Bound
96-107	LSVYKPKDIVDP(ENL)	1729.9	3.82	0	6.3 ± 0.1	6.3 ± 0.1	6.0 ± 0.1	Free
			3.85	0	11.1 ± 0.1	11 ± 0.1	11.5 ± 0	Bound
104-107	IVDP(ENL)	799.4	3.85	0	2.4 ± 0.03	2.4 ± 0.01	2.18 ± 0.09	Free
			3.86	0	4 ± 0.1	4.07 ± 0.01	4.33 ± 0.01	Bound
108-116	(YFQG)ATPYPGDKV	1442.7	3.60	0	5.1 ± 0.1	5 ± 0.1	4.6 ± 0.1	Free
			3.58	0	7.6 ± 0.3	8.4 ± 0.2	9.1 ± 0.3	Bound
108-120	(YFQG)ATPYPGDKVIITE	1899.0	3.84	0	7.3 ± 0.2	7.2 ± 0.1	6.7 ± 0.2	Free
			3.87	0	12.6 ± 0.5	12.7 ± 0.2	13.6 ± 0.2	Bound
121-129	GAFEGFQAI	939.5	4.41	0	4.5 ± 0.1	4.49 ± 0.02	4.23 ± 0.08	Free
			4.51	0	7.11 ± 0.07	7.89 ± 0.07	8.01 ± 0.07	Bound
130-141	FTEPDGEARSML	1352.6	3.80	0	3.4 ± 0.1	3.7 ± 0.1	3.9 ± 0.1	Free
			3.80	0	5.1 ± 0.3	5.9 ± 0.2	7 ± 0.4	Bound
142-159	LLNLINKEIKHSVKNTEF	2140.2	3.72	0	7.4 ± 0.1	7.4 ± 0.1	6.9 ± 0.1	Free
			3.72	0	11.1 ± 0.1	12 ± 0.1	12.9 ± 0.1	Bound
146-159	INKEIKHSVKNTEF	1686.9	2.50	0	5.5 ± 0.3	5.6 ± 0.1	5.2 ± 0.2	Free
			2.72	0	9.5 ± 0.3	9.4 ± 0.2	10 ± 0.2	Bound

Expressed as average ± std. error of triplicates.
tr(0): Retention time.
(ENL) and (YFQG) correspond to the TEV cleaving sequence and was not considered in the sequence numbering.

Supplementary Table S9. Deuteron incorporation for NusG under different reaction times.

Position	Sequence	Mass, Da	tr(0), min	Deuteron incorporation, AMU ± SD				State
				0 min	0.5 min	1.0 min	2.0 min	
2-14	SEAPKKRWYVVQA	1561.8	3.24	0	4.3 ± 0.2	4.8 ± 0.2	5.9 ± 0.1	Free
			3.22	0	3.7 ± 0.1	4 ± 0.1	4.6 ± 0.1	Bound
14-28	AFSGFEGRVATSL	1341.7	4.10	0	8.2 ± 0.3	9.2 ± 0.2	9.8 ± 0.08	Free
			4.05	0	7.4 ± 0.1	8.44 ± 0.07	9.3 ± 0.08	Bound
29-38	HIKLHNMEDL	1249.6	3.20	0	3.6 ± 0.1	3.9 ± 0.1	4.4 ± 0.1	Free
			3.31	0	3.3 ± 0.1	4 ± 0.2	4.2 ± 0.3	Bound
41-48	EVMVPTEE	933.4	3.25	0	4.4 ± 0.4	4.5 ± 0.2	4.8 ± 0.1	Free
			3.25	0	4.5 ± 0.1	4.6 ± 0.2	4.6 ± 0.2	Bound
49-70	VVEIRGGQRRKSERKFFPGYVL	2621.5	3.48	0	10.6 ± 0.9	10.4 ± 0.7	11.2 ± 0.1	Free
			3.54	0	11.5 ± 0.5	11.7 ± 0.7	12.2 ± 0.4	Bound
71-80	VQMVMNDASW	1180.5	4.39	0	3.4 ± 0.3	3.8 ± 0.1	4.3 ± 0.1	Free
			4.34	0	3.4 ± 0.1	3.6 ± 0.1	4.1 ± 0.1	Bound
74-80	VMNDASW	822.3	3.94	0	2.11 ± 0.08	2.41 ± 0.06	2.89 ± 0.01	Free
			3.92	0	2.1 ± 0.05	2.32 ± 0.06	2.65 ± 0.08	Bound
81-109	HLVRSVPRVMGFIGGTSDRPAPISDKEVD	3135.6	3.45	0	19 ± 1	19.8 ± 0.7	20.4 ± 0.2	Free
			3.42	0	18.2 ± 0.2	19.2 ± 0.6	20.0 ± 0.2	Bound
92-109	FIGGTSDRPAPISDKEVD	1903.9	3.07	0	12.4 ± 0.7	12.5 ± 0.5	12.8 ± 0.1	Free
			3.08	0	10.7 ± 0.1	11.0 ± 0.4	11.5 ± 0.1	Bound
110-133	AIMNRLQQVGDKPRPKTLFEPGEM	2755.4	3.62	0	18 ± 1	18 ± 1	19.5 ± 0.2	Free
			3.57	0	18.5 ± 0.5	19 ± 1	19.2 ± 0.2	Bound
134-144	VRVNDGPFADF	1236.6	4.57	0	5.2 ± 0.1	5.7 ± 0.1	6.3 ± 0.1	Free
			4.51	0	5.0 ± 0.2	5.6 ± 0.2	6.3 ± 0.2	Bound
134-150	VRVNDGPFADFNGVVEE	1863.9	4.40	0	7.2 ± 0.2	7.9 ± 0.1	8.9 ± 0.1	Free
			4.34	0	6.9 ± 0.1	7.9 ± 0.1	8.6 ± 0.1	Bound
151-158	VDYEKSRL	1009.5	2.82	0	4.6 ± 0.2	4.8 ± 0.1	5.26 ± 0.07	Free
			2.66	0	4.55 ± 0.06	4.8 ± 0.1	5.12 ± 0.05	Bound
159-165	KVSVSIF	779.5	4.06	0	4.4 ± 0.2	4.7 ± 0.2	5.25 ± 0.02	Free
			4.02	0	4.29 ± 0.05	4.76 ± 0.02	5.01 ± 0.02	Bound
159-174	KVSVSIFGRATPVELD	1717.9	4.12	0	10.8 ± 0.6	11.2 ± 0.5	12.3 ± 0.1	Free
			4.07	0	10.3 ± 0.1	10.6 ± 0.1	10.5 ± 0	Bound
174-181	DFSQVEKA	923.4	3.05	0	4.1 ± 0.1	4.3 ± 0.1	4.9 ± 0	Free
			3.04	0	3.74 ± 0.03	4.01 ± 0.06	4.42 ± 0.05	Bound

Expressed as average ± std. error of fit
tr(0): Retention time.

Supplementary Table S10. Deuteron incorporation for isolated CTD of RfaH under different reaction times.

Position	Sequence	Mass, Da	tr(0), min	Deuteron incorporation, AMU ± SD				State
				0 min	0.5 min	1.0 min	2.0 min	
117-123	IITEGAF	750.4	3.98	0	3.8 ± 0.1	4 ± 0.1	3.9 ± 0.1	Free
124-142	EGFQAIFTEPDGEARSMLL	2111	5.02	0	11.6 ± 0.7	12.6 ± 0.8	12 ± 1	Free
130-142	FTEPDGEARSMLL	1465.7	4.22	0	6.13 ± 0.04	6.7 ± 0.2	6.5 ± 0.3	Free
143-158	LNLINKEIKHSVKNTE	1880.1	2.83	0	9.4 ± 0.2	10.1 ± 0.4	9.6 ± 0.5	Free
146-158	INKEIKHSVKNTE	1539.8	1.63	0	6.9 ± 0.1	7.3 ± 0.3	7.0 ± 0.3	Free
146-159	INKEIKHSVKNTEF	1686.9	2.49	0	6.8 ± 0.2	7.3 ± 0.3	7.0 ± 0.4	Free

Expressed as average ± std. error of fit
tr(0): Retention time.

Supplementary Table S11. Deuteron incorporation for RNAP β subunit upon NusG binding under different reaction times.

Position	Sequence	Mass, Da	tr(0), min	Deuteron incorporation, AMU ± SD				State
				0 min	0.5 min	1.0 min	2.0 min	
53-68	FRSVFPIQSYSGNSEL	1830.9	4.40	0	6.41 ± 0.07	6.81 ± 0.08	6.6 ± 0.2	Free
			4.38	0	5.8 ± 0.1	6.1 ± 0.2	6.5 ± 0.1	Bound
69-80	QYVSYRLGEPVF	1457.7	4.37	0	5.07 ± 0.01	5.77 ± 0.09	6.18 ± 0.04	Free
			4.35	0	5.4 ± 0	6 ± 0.2	6.3 ± 0.2	Bound
81-102	DVQECQIRGVTYSAPLRVKLRL	2544.4	3.81	0	6.3 ± 0.2	7.1 ± 0.1	7.5 ± 0.2	Free
			3.79	0	7 ± 1	6.9 ± 0.1	7.3 ± 0.2	Bound
103-121	VIYEREAPEGTVKDIKEQE	2233.1	2.91	0	14.66 ± 0.08	14.95 ± 0.08	14.6 ± 0.2	Free
			2.90	0	14.0 ± 0.1	14.5 ± 0.1	14.4 ± 0.1	Bound
120-136	QEVYMGEIPLMTDNGTF	1944.9	4.98	0	3.8 ± 0.2	4.1 ± 0.1	4.1 ± 0.2	Free
			4.96	0	3.7 ± 0.1	3.8 ± 0.2	3.8 ± 0.1	Bound
137-149	VINGTERVIVSQL	1427.8	3.89	0	1.48 ± 0.04	1.79 ± 0.09	1.99 ± 0.07	Free
			3.87	0	1.7 ± 0.3	1.9 ± 0.2	2 ± 0.1	Bound
157-183	FDSDKGKTHSSGKVLYNARIIPYRGSW	3082.6	3.50	0	6.9 ± 0.2	7 .0± 0.3	7.3 ± 0.3	Free
			3.48	0	7.3 ± 0.1	7.7 ± 0.2	7.6 ± 0.1	Bound
187-195	EFDPKDNLF	1124.5	4.22	0	1.38 ± 0.06	1.53 ± 0.08	1.6 ± 0.1	Free
			4.17	0	1.6 ± 0.1	1.8 ± 0.1	1.87 ± 0.08	Bound
211-223	RALNYTTEQILDL	1549.8	4.41	0	2.79 ± 0.02	2.89 ± 0.02	3.03 ± 0.04	Free
			4.40	0	2.93 ± 0.08	3.00 ± 0.09	3.15 ± 0.09	Bound
224-230	FFEKVIF	929.5	4.57	0	1.89 ± 0.06	2.62 ± 0.05	3.09 ± 0.05	Free
			4.55	0	2.08 ± 0.03	2.81 ± 0.06	3.22 ± 0.03	Bound
231-239	EIRDNKLQM	1146.6	3.04	0	3.1 ± 0.2	3.6 ± 0.1	4.7 ± 0.1	Free
			3.02	0	3.9 ± 0.1	4.2 ± 0	4.3 ± 0.1	Bound
240-253	ELVPERLRGETASF	1603.8	3.66	0	6.11 ± 0.03	6.84 ± 0.06	7.4 ± 0.1	Free
			3.63	0	6.3 ± 0.1	6.8 ± 0.2	7.69 ± 0.04	Bound
322-336	LAKLSQSGHKRIETL	1681.0	1.75	0	3.2 ± 0.5	3.7 ± 0.1	4.2 ± 0.4	Free
			1.76	0	3.4 ± 0.6	3.9 ± 0.3	4.4 ± 0.5	Bound
337-349	FTNDLDHGPYISE	1507.7	3.65	0	4.9 ± 0.1	5.2 ± 0.1	5.1 ± 0.1	Free
			3.63	0	4.7 ± 0.2	4.9 ± 0.2	4.8 ± 0.2	Bound
351-363	LRVDPTNDRLSAL	1469.8	3.55	0	4.89 ± 0.04	5.0 ± 0.2	5.20 ± 0.02	Free
			3.54	0	4.7 ± 0.1	4.70 ± 0.07	4.8 ± 0.1	Bound
364-384	VEIYRMMRPGEPPTREAAESL	2432.2	3.60	0	8.5 ± 0.3	9.3 ± 0.2	9.7 ± 0.4	Free
			3.58	0	6.98 ± 0.09	7.7 ± 0.2	8.5 ± 0.1	Bound
400-412	VGRMKFNRSLLRE	1605.9	3.08	0	2.61 ± 0.08	3.24 ± 0.08	3.97 ± 0.09	Free
			3.06	0	2.9 ± 0.2	3.19 ± 0.05	3.99 ± 0.05	Bound
413-428	EIEGSGILSKDDIIDV	1702.9	4.28	0	5.67 ± 0.06	5.72 ± 0.05	5.64 ± 0.05	Free
			4.27	0	5.50 ± 0.03	5.5 ± 0.2	5.47 ± 0.07	Bound
460-468	AENQFRVGL	1033.5	3.81	0	3.8 ± 0.1	3.9 ± 0.1	4.0 ± 0.1	Free
			3.95	0	5.1 ± 0.05	5.0 ± 0.2	5.21 ± 0.06	Bound
485-500	DTLMPQDMINAKPISA	1744.9	3.93	0	8.4 ± 0.2	9.7 ± 0.1	10.7 ± 0.1	Free
			3.92	0	8.6 ± 0.1	9.5 ± 0.2	10.5 ± 0.1	Bound
501-514	AVKEFFGSSQLSQF	1574.8	4.35	0	5.76 ± 0.04	6.21 ± 0.04	6.8 ± 0.1	Free
			4.33	0	5.58 ± 0.07	6.0 ± 0.1	6.4 ± 0.1	Bound

546-574	EVRDVHPTHYGRVCPIETPEGPNIGLINS	3199.6	3.66	0	3.2 ± 0.3	3.4 ± 0.3	3.6 ± 0.2	Free
			3.63	0	3.5 ± 0.4	4.0 ± 0.2	3.9 ± 0.3	Bound
587-608	LETPYRKVTDGVVTDEIHYLSA	2506.3	4.08	0	6.8 ± 0.3	7.4 ± 0.3	7.1 ± 0.6	Free
			4.05	0	6.8 ± 0.1	7.1 ± 0.1	7.2 ± 0.1	Bound
624-632	DEEGHFVED	1076.4	3.03	0	3.7 ± 0.1	4.5 ± 0.1	5.1 ± 0.2	Free
			3.02	0	3.6 ± 0.1	4.4 ± 0.1	5.0 ± 0.1	Bound
645-652	FSRDQVDY	1029.5	3.16	0	1.8 ± 0.1	2.01 ± 0.05	2.13 ± 0.04	Free
			3.14	0	1.7 ± 0.1	1.93 ± 0.06	2.1 ± 0.1	Bound
665-681	ASLIPFLEHDDANRALM	1913.0	4.48	0	2.49 ± 0.09	2.5 ± 0.08	2.41 ± 0.07	Free
			4.46	0	2.6 ± 0.07	2.7 ± 0.5	2.41 ± 0.05	Bound
686-693	QRQAVPTL	912.5	3.31	0	0.57 ± 0.03	0.6 ± 0.1	0.64 ± 0.08	Free
			3.29	0	0.7 ± 0.1	0.64 ± 0.02	0.76 ± 0.03	Bound
694-707	RADKPLVGTGMERA	1500.8	1.76	0	4.1 ± 0.3	4.5 ± 0.2	4.4 ± 0.2	Free
			1.78	0	4.6 ± 0.3	4.6 ± 0.3	4.1 ± 0.2	Bound
708-716	VAVDSGVTA	818.4	2.76	0	2.13 ± 0.07	2.35 ± 0.07	2.77 ± 0.06	Free
			2.72	0	2.3 ± 0.2	2.6 ± 0.2	2.84 ± 0.05	Bound
730-749	SRIVIKVNEDEMYPGEAGID	2235.1	3.68	0	3.3 ± 0.1	3.9 ± 0.2	4.5 ± 0.2	Free
			3.67	0	4.3 ± 0.4	4.4 ± 0.1	4.7 ± 0.1	Bound
750-772	IYNLTKYTRSNQNTCINQMPCVS	2691.3	3.67	0	3.3 ± 0.1	3.9 ± 0.2	4.5 ± 0.2	Free
			3.66	0	4.3 ± 0.4	4.4 ± 0.1	4.7 ± 0.1	Bound
773-783	LGEPVERGDVL	1183.6	3.56	0	1.05 ± 0.07	1.44 ± 0.09	1.97 ± 0.08	Free
			3.54	0	1.1 ± 0.1	1.5 ± 0.1	1.9 ± 0.1	Bound
800-811	MRVAFMPWNGYN	1485.7	4.54	0	1.73 ± 0.04	2.08 ± 0.08	2.49 ± 0.05	Free
			4.53	0	2 ± 0.2	2.3 ± 0.2	2.61 ± 0.03	Bound
964-971	LQILEAGL	856.5	4.44	0	1.96 ± 0.06	2.3 ± 0.1	2.89 ± 0.04	Free
			4.41	0	1.8 ± 0.2	2.1 ± 0.2	2.6 ± 0.2	Bound
972-979	FSRIRAVL	961.6	3.44	0	0.48 ± 0.05	0.7 ± 0.06	1.02 ± 0.05	Free
			3.43	0	0.49 ± 0.05	0.73 ± 0.05	1.05 ± 0.05	Bound
998-1014	LELGLTDEEKQNQLEQL	2000.0	4.06	0	11.3 ± 0.3	12.3 ± 0.2	12.8 ± 0.3	Free
			4.05	0	11.1 ± 0.1	11.9 ± 0.2	12.6 ± 0.2	Bound
1048-1054	KIVKVYL	862.6	3.40	0	0.01 ± 0.07	0.01 ± 0.06	0.05 ± 0.07	Free
			3.39	0	0.05 ± 0.04	0.07 ± 0.05	0.05 ± 0.04	Bound
1096-1107	IVLNPLGVPSRM	1295.8	4.19	0	5.80 ± 0.04	6.03 ± 0.03	5.96 ± 0.08	Free
			4.17	0	5.71 ± 0.08	5.86 ± 0.03	5.75 ± 0.08	Bound
1108-1115	NIGQILET	887.5	4.00	0	0.56 ± 0.01	0.7 ± 0.1	0.53 ± 0.03	Free
			3.98	0	0.7 ± 0.2	0.7 ± 0.2	0.5 ± 0.04	Bound
1131-1143	MLKQQQEVAKLRE	1600.9	1.76	0	9.00 ± 0.09	9.29 ± 0.07	9.6 ± 0.1	Free
			1.77	0	8.9 ± 0.1	9.1 ± 0.3	9.6 ± 0.1	Bound
1144-1153	FIQRAYDLGA	1153.6	3.52	0	2.5 ± 0.1	2.5 ± 0.1	2.6 ± 0.2	Free
			3.51	0	2.42 ± 0.06	2.47 ± 0.06	2.47 ± 0.06	Bound
1173-1184	AENLRKGMPIAT	1300.7	2.93	0	1.75 ± 0.04	1.89 ± 0.08	1.8 ± 0.1	Free
			2.91	0	1.9 ± 0.2	2.0 ± 0.2	2.0 ± 0.3	Bound
1185-1194	PVFDGAKEAE	1062.5	2.94	0	3.32 ± 0.08	3.58 ± 0.06	3.95 ± 0.07	Free
			2.92	0	3.5 ± 0.2	3.64 ± 0.08	3.89 ± 0.04	Bound
1198-1212	LLKLGDLPTSGQIRL	1624.0	4.20	0	5.09 ± 0.04	5.27 ± 0.07	5.29 ± 0.05	Free
			4.18	0	5.1 ± 0.2	5.1 ± 0.1	5.12 ± 0.05	Bound

1213-1220	YDGRTGEQ	925.4	1.70	0	1.9 ± 0.1	1.9 ± 0.1	1.9 ± 0.1	Free
			1.71	0	2.1 ± 0.1	2.1 ± 0.2	2.1 ± 0.1	Bound
1221-1229	FERPVTVGY	1067.6	3.41	0	0.3 ± 0.1	0.3 ± 0.1	0.3 ± 0.1	Free
			3.40	0	0.4 ± 0.2	0.3 ± 0.2	0.3 ± 0.2	Bound
1291-1304	LTVKSDDVNGRTKM	1563.8	1.74	0	5.9 ± 0.8	5.8 ± 0.9	6.4 ± 0.8	Free
			1.75	0	6.3 ± 0.9	6.3 ± 0.8	6.4 ± 0.8	Bound
1305-1323	YKNIVDGNHQMEPGMPESF	2193.0	3.77	0	5.43 ± 0.09	5.6 ± 0.1	5.73 ± 0.06	Free
			3.75	0	4.8 ± 0.3	4.73 ± 0.02	4.47 ± 0.03	Bound
1327-1336	LKEIRSLGIN	1142.7	3.23	0	0.29 ± 0.03	0.29 ± 0.02	0.26 ± 0.02	Free
			3.21	0	0.4 ± 0.1	0.5 ± 0.3	0.3 ± 0.03	Bound

Expressed as average ± std. error of fit
tr(0): Retention time.

Supplementary Table S12. Deuteron incorporation for RNAP β subunit upon RfaH binding under different reaction times.

Position	Sequence	Mass, Da	tr(0), min	Deuteron incorporation, AMU ± SD				State
				0 min	0.5 min	1.0 min	2.0 min	
39-52	IEQDPEGQYGLEAA	1519.7	3.61	0	4.6 ± 0.2	5.3 ± 0.1	5.7 ± 0.5	Free
			3.77	0	5.3 ± 0.1	5.8 ± 0.1	6.5 ± 0.1	Bound
53-68	FRSVFPIQSYSGNSEL	1830.9	4.40	0	6.41 ± 0.07	6.81 ± 0.08	6.6 ± 0.2	Free
			4.30	0	6.5 ± 0.1	6.8 ± 0.1	7.1 ± 0.4	Bound
69-80	QYVSYRLGEPVF	1457.7	4.37	0	5.07 ± 0.01	5.77 ± 0.09	6.18 ± 0.04	Free
			4.28	0	5.37 ± 0.08	6.1 ± 0.1	6.87 ± 0.07	Bound
103-121	VIYEREAPEGTVKDIKEQE	2233.1	2.91	0	14.66 ± 0.08	14.95 ± 0.08	14.6 ± 0.2	Free
			3.10	0	14.2 ± 0.3	14.6 ± 0.2	15.4 ± 0.2	Bound
150-156	HRSPGVF	799.4	2.90	0	0.65 ± 0.06	0.83 ± 0.07	1.11 ± 0.06	Free
			3.08	0	0.9 ± 0.1	1.0 ± 0.1	1.0 ± 0.1	Bound
175-183	RIIPYRGSW	1147.6	3.64	0	1.54 ± 0.06	1.63 ± 0.07	1.65 ± 0.05	Free
			3.7	0	1.79 ± 0.09	1.86 ± 0.08	1.94 ± 0.04	Bound
187-194	EFDPKDNL	977.5	3.44	0	1.36 ± 0.07	1.55 ± 0.06	1.51 ± 0.05	Free
			3.58	0	1.7 ± 0.2	1.7 ± 0.2	1.7 ± 0.2	Bound
224-230	FFEKVIF	929.5	4.57	0	1.89 ± 0.06	2.62 ± 0.05	3.09 ± 0.05	Free
			4.42	0	2.3 ± 0.1	2.95 ± 0.03	3.45 ± 0.05	Bound
231-239	EIRDNKLQM	1146.6	3.04	0	3.1 ± 0.2	3.6 ± 0.1	4.7 ± 0.1	Free
			3.20	0	3.9 ± 0.1	4.2 ± 0.1	4.5 ± 0.1	Bound
240-253	ELVPERLRGETASF	1603.8	3.65	0	6.11 ± 0.03	6.84 ± 0.06	7.4 ± 0.1	Free
			3.74	0	6.4 ± 0.1	7.0 ± 0.2	7.76 ± 0.04	Bound
285-291	IEVPVEY	848.4	3.86	0	2.23 ± 0.08	2.55 ± 0.07	2.65 ± 0.07	Free
			3.95	0	2.42 ± 0.13	2.47 ± 0.03	2.74 ± 0.05	Bound
337-349	FTNDLDHGPYISE	1507.7	3.65	0	4.9 ± 0.1	5.2 ± 0.1	5.1 ± 0.1	Free
			3.75	0	5.42 ± 0.05	5.41 ± 0.06	5.51 ± 0.02	Bound
351-360	LRVDPTNDRL	1198.7	3.14	0	4.2 ± 0.1	4.1 ± 0.1	4.2 ± 0.1	Free
			3.29	0	3.7 ± 0.2	3.8 ± 0.2	4.0 ± 0.2	Bound
364-384	VEIYRMMRPGEPPTREAAESL	2432.2	3.60	0	8.5 ± 0.3	9.3 ± 0.2	9.7 ± 0.4	Free
			3.68	0	6.6 ± 0.1	7.07 ± 0.08	7.4 ± 0.2	Bound
390-399	FSEDRYDLSA	1202.5	3.43	0	3.67 ± 0.07	3.65 ± 0.05	3.5 ± 0.05	Free
			3.56	0	3.9 ± 0.1	3.9 ± 0.3	3.8 ± 0.1	Bound
400-410	VGRMKFNRSLL	1320.8	3.42	0	1.42 ± 0.06	1.9 ± 0.06	2.62 ± 0.08	Free
			3.52	0	2.4 ± 0.2	3.2 ± 0.4	3.2 ± 0.1	Bound
460-468	AENQFRVGL	1033.5	3.81	0	3.8 ± 0.1	3.9 ± 0.1	4.0 ± 0.1	Free
			3.89	0	3.0 ± 0.2	3.05 ± 0.01	3.09 ± 0.03	Bound
501-511	AVKEFFGSSQL	1212.6	4.06	0	4.7 ± 0.1	5.2 ± 0.1	5.9 ± 0.2	Free
			4.05	0	5.4 ± 0.2	6.1 ± 0.2	6.6 ± 0.3	Bound
624-632	DEEGHFVED	1076.4	3.03	0	3.7 ± 0.1	4.5 ± 0.1	5.1 ± 0.2	Free
			3.25	0	4.1 ± 0.1	4.9 ± 0.1	5.3 ± 0.1	Bound
645-653	FSRDQVDYM	1913.0	4.48	0	1.8 ± 0.1	2.01 ± 0.05	2.13 ± 0.04	Free
			4.36	0	1.88 ± 0.04	2.16 ± 0.08	2.28 ± 0.09	Bound
686-693	QRQAVPTL	912.5	3.31	0	0.57 ± 0.03	0.6 ± 0.1	0.64 ± 0.08	Free
			3.43	0	1.4 ± 0.1	1.4 ± 0.2	1.19 ± 0.04	Bound
694-709	RADKPLVGTGMERAVA	1670.9	2.95	0	4.2 ± 0.1	4.5 ± 0.1	4.3 ± 0.1	Free
			3.13	0	4.9 ± 0.1	5.0 ± 0.3	4.7 ± 0.2	Bound
708-716	VAVDSGVTA	818.4	2.76	0	2.13 ± 0.07	2.35 ± 0.07	2.77 ± 0.06	Free
			3.06	0	2.3 ± 0.2	2.83 ± 0.05	3.0 ± 0.3	Bound

717-729	VAKRGGVVQYVDA	1361.8	3.06	0	2.7 ± 0.3	2.9 ± 0.3	3.4 ± 0.4	Free
			3.14	0	2.7 ± 0.2	2.8 ± 0.1	3.0 ± 0.1	Bound
750-772	IYNLTKYTRSNQNTCINQMPCVS	2691.3	3.67	0	3.3 ± 0.1	3.9 ± 0.2	4.5 ± 0.2	Free
			3.77	0	4.6 ± 0.4	5.3 ± 0.5	5.2 ± 0.2	Bound
876-883	EVTGGDIL	803.4	3.88	0	2.8 ± 0.2	3.2 ± 0.01	3.31 ± 0.04	Free
			3.99	0	3.0 ± 0.1	3.29 ± 0.02	3.39 ± 0.04	Bound
951-964	MQLKQAKKDLSEEL	1660.9	3.35	0	4.5 ± 0.1	6.03 ± 0.08	7.7 ± 0.1	Free
			3.48	0	5.0 ± 0.2	6.6 ± 0.2	8.3 ± 0.2	Bound
965-971	QILEAGL	743.4	3.96	0	1.94 ± 0.03	2.19 ± 0.07	2.48 ± 0.09	Free
			4.03	0	1.83 ± 0.05	2.09 ± 0.03	2.55 ± 0.04	Bound
972-979	FSRIRAVL	961.6	3.44	0	0.48 ± 0.05	0.7 ± 0.06	1.02 ± 0.05	Free
			3.54	0	0.58 ± 0.06	0.8 ± 0.07	1.14 ± 0.06	Bound
1048-1054	KIVKVYL	862.6	3.40	0	0.01 ± 0.07	0.01 ± 0.06	0.05 ± 0.07	Free
			3.50	0	0.01 ± 0.06	0.06 ± 0.08	0.02 ± 0.06	Bound
1096-1115	IVLNPLGVPSRMNIGQILET	2164.2	4.73	0	6.0 ± 0.1	6.3 ± 0.1	6.2 ± 0.1	Free
			4.55	0	7.4 ± 0.4	7.5 ± 0.5	6.7 ± 0.2	Bound
1144-1153	FIQRAYDLGA	1153.6	3.52	0	2.5 ± 0.1	2.5 ± 0.1	2.6 ± 0.2	Free
			3.62	0	2.7 ± 0.2	2.8 ± 0.2	2.8 ± 0.2	Bound
1173-1184	AENLRKGMPIAT	1300.7	2.93	0	1.75 ± 0.04	1.89 ± 0.08	1.8 ± 0.1	Free
			3.11	0	2.1 ± 0.3	1.9 ± 0.1	1.88 ± 0.06	Bound
1185-1194	PVFDGAKEAE	1062.5	2.94	0	3.32 ± 0.08	3.58 ± 0.06	3.95 ± 0.07	Free
			3.15	0	4.1 ± 0.1	4.4 ± 0.2	4.5 ± 0.1	Bound
1198-1212	LLKLGDLPTSGQIRL	1624.0	4.20	0	5.09 ± 0.04	5.27 ± 0.07	5.29 ± 0.05	Free
			4.15	0	5.88 ± 0.08	6.1 ± 0.1	5.71 ± 0.07	Bound
1221-1229	FERPVTVGY	1067.6	3.41	0	0.3 ± 0.1	0.3 ± 0.1	0.3 ± 0.1	Free
			3.53	0	1.2 ± 0.3	1.2 ± 0.3	1.0 ± 0.3	Bound
1254-1273	VTQQPLGGKAQFGGQRFGEM	2136.1	3.67	0	8.53 ± 0.09	8.9 ± 0.2	9.3 ± 0.1	Free
			3.74	0	9.1 ± 0.2	9.3 ± 0.2	9.5 ± 0.1	Bound
1327-1336	LKEIRSLGIN	1142.7	3.23	0	0.29 ± 0.03	0.29 ± 0.02	0.26 ± 0.02	Free
			3.36	0	1.9 ± 0.3	1.8 ± 0.3	1.1 ± 0.1	Bound

Expressed as average ± std. error of fit
tr(0): Retention time.

Supplementary Table S13. Deuteron incorporation for RNAP β' subunit upon NusG binding under different reaction times.

Position	Sequence	Mass, Da	tr(0), min	Deuteron incorporation, AMU ± SD				State
				0 min	0.5 min	1.0 min	2.0 min	
89-99	LKSLPSRIGLL	1196.8	3.93	0	3.7 ± 0.05	3.8 ± 0.1	4.05 ± 0.06	Free
			3.90	0	3.62 ± 0.08	3.77 ± 0.07	3.96 ± 0.05	Bound
100-112	LDMPLRDIERVLY	1632.9	4.71	0	2.7 ± 0.2	2.8 ± 0.2	2.8 ± 0.3	Free
			4.69	0	2.8 ± 0.1	2.8 ± 0.2	2.9 ± 0.1	Bound
127-136	ERQQILTEEQ	1273.6	2.91	0	1.9 ± 0.1	1.9 ± 0.2	1.9 ± 0.4	Free
			2.88	0	1.9 ± 0.3	2.0 ± 0.8	2.0 ± 0.2	Bound
141-148	LEEFGDEF	838.3	3.55	0	2.23 ± 0.05	2.64 ± 0.02	3.13 ± 0.05	Free
			3.55	0	2.22 ± 0.06	2.59 ± 0.06	3.10 ± 0.05	Bound
212-232	TVLPVLPPDLRPLVPLDGGRF	2271.3	4.74	0	9.00 ± 0.03	9.53 ± 0.04	9.7 ± 0.1	Free
			4.71	0	7.42 ± 0.04	7.97 ± 0.04	8.19 ± 0.07	Bound
260-273	PDIIVRNEKRMLQE	1740.9	3.28	0	5.4 ± 0.1	6.3 ± 0.2	6.2 ± 0.1	Free
			3.26	0	2.2 ± 0.1	2.4 ± 0.4	2.51 ± 0.08	Bound
326-335	VITVGPYLRL	1130.7	4.55	0	1.00 ± 0.08	1.26 ± 0.06	1.5 ± 0.1	Free
			4.53	0	1.3 ± 0.2	1.44 ± 0.04	1.51 ± 0.03	Bound
347-359	ELFKPFIYGKLEL	1596.9	4.69	0	1.59 ± 0.04	2.30 ± 0.06	3.17 ± 0.05	Free
			4.67	0	1.9 ± 0.2	2.7 ± 0.2	3.44 ± 0.04	Bound
379-386	VVWDILDE	988.5	5.00	0	0.99 ± 0.08	1.26 ± 0.06	1.51 ± 0.05	Free
			4.98	0	1.22 ± 0.06	1.46 ± 0.06	1.84 ± 0.04	Bound
395-413	LNRAPTLHRLGIQAFEPVL	1836.0	3.71	0	2.42 ± 0.05	2.72 ± 0.05	3.01 ± 0.03	Free
			3.69	0	2.8 ± 0.2	3.4 ± 0.7	3.09 ± 0.04	Bound
414-426	IEGKAIQLHPLVC	1420.8	3.69	0	2.39 ± 0.05	2.35 ± 0.05	2.37 ± 0.04	Free
			3.67	0	2.6 ± 0.2	2.5 ± 0.3	2.4 ± 0.1	Bound
439-446	AVHVPLTL	849.5	3.97	0	0.71 ± 0.06	0.86 ± 0.08	1.0 ± 0.1	Free
			3.94	0	0.6 ± 0.1	0.43 ± 0.03	0.35 ± 0.04	Bound
581-592	YRILGLKPTVIF	1419.9	4.40	0	2.3 ± 0.02	2.38 ± 0.02	2.31 ± 0.04	Free
			4.38	0	2.4 ± 0.04	2.40 ± 0.1	2.35 ± 0.06	Bound
616-628	MVIPEKKHEIISE	1552.8	2.84	0	3.22 ± 0.07	3.52 ± 0.09	4.0 ± 0.2	Free
			2.79	0	3.4 ± 0.2	3.6 ± 0.3	3.99 ± 0.07	Bound
745-760	FISTHGARKGLADTAL	1657.9	3.12	0	4.1 ± 0.2	4.8 ± 0.2	5.5 ± 0.3	Free
			3.11	0	4.7 ± 0.3	5.6 ± 0.7	5.8 ± 0.1	Bound
796-818	PVIEGGDVKEPLRDRVLGRVTAE	2505.4	3.34	0	10.62 ± 0.08	11.16 ± 0.07	11.4 ± 0.1	Free
			3.33	0	10.5 ± 0.2	11.1 ± 0.3	11.2 ± 0.1	Bound
828-836	ILVPRNTLL	1038.7	4.00	0	1.90 ± 0.06	1.95 ± 0.09	2.00 ± 0.07	Free
			3.98	0	2.2 ± 0.3	2.1 ± 0.4	2.1 ± 0.1	Bound
875-891	LARGHIINKGEAIGVIA	1732.0	3.18	0	3.2 ± 0.1	3.2 ± 0.4	3.2 ± 0.1	Free
			3.16	0	3.4 ± 0.2	3.4 ± 0.1	3.3 ± 0.1	Bound
903-921	TMRTFHIGGAASRAAAESS	1920.9	3.03	0	15.5 ± 0.1	15.5 ± 0.1	15.0 ± 0.3	Free
			3.02	0	14.2 ± 0.5	14.5 ± 0.7	14.7 ± 0.2	Bound
946-956	VITSRNTELKL	1273.7	3.35	0	3.4 ± 0.1	3.5 ± 0.1	3.9 ± 0.2	Free
			3.34	0	3.3 ± 0.1	3.6 ± 0.2	3.7 ± 0.1	Bound
960-975	FGRTKESYKVPYGAVL	1815.0	3.58	0	3.8 ± 0.1	4.2 ± 0.1	4.4 ± 0.2	Free
			3.55	0	4.0 ± 0.1	4.3 ± 0.2	4.5 ± 0.1	Bound
1087-1093	ISSGDTL	692.3	3.11	0	2.30 ± 0.01	2.29 ± 0.03	2.2 ± 0.02	Free
			3.10	0	2.18 ± 0.06	2.16 ± 0.01	2.06 ± 0.01	Bound
1094-1116	ARIPQESGGTKDITGGLPRVADL	2351.3	3.75	0	11.6 ± 0.1	12.02 ± 0.04	12.1 ± 0.1	Free
			3.73	0	11.2 ± 0.2	11.7 ± 0.1	11.9 ± 0.1	Bound

1117-1128	FEARRPKEPAIL	1426.8	3.13	0	4.4 ± 0.1	4.73 ± 0.09	4.81 ± 0.03	Free
			3.12	0	4.4 ± 0.1	4.69 ± 0.09	4.7 ± 0.1	Bound
1129-1137	AEISGIVSF	922.5	4.48	0	0.72 ± 0.04	0.97 ± 0.02	1.45 ± 0.03	Free
			4.46	0	0.67 ± 0.06	1.02 ± 0.06	1.4 ± 0.02	Bound
1209-1215	VQDVYRL	892.5	3.65	0	1.34 ± 0.07	1.60 ± 0.08	1.48 ± 0.07	Free
			3.63	0	1.37 ± 0.22	1.77 ± 0.27	1.69 ± 0.06	Bound
1298-1306	QETTRVLTE	1076.6	2.68	0	2.82 ± 0.04	2.87 ± 0.05	2.89 ± 0.04	Free
			2.63	0	2.6 ± 0.1	2.72 ± 0.06	2.70 ± 0.08	Bound

Expressed as average ± std. error of fit
tr(0): Retention time.

Supplementary Table S14. Deuteron incorporation for RNAP β' subunit upon RfaH binding under different reaction times.

Position	Sequence	Mass, Da	tr(0), min	Deuteron incorporation, AMU ± SD				State
				0 min	0.5 min	1.0 min	2.0 min	
90-100	KSLPSRIGLLL	1196.8	3.93	0	3.7 ± 0.05	3.8 ± 0.1	4.05 ± 0.06	Free
			3.93	0	3.89 ± 0.08	4.00 ± 0.03	4.32 ± 0.08	Bound
100-108	LDMPLRDIE	1101.6	3.95	0	2.5 ± 0.2	2.76 ± 0.03	2.73 ± 0.05	Free
			3.99	0	2.67 ± 0.09	2.69 ± 0.07	2.79 ± 0.09	Bound
127-136	ERQQILTEEQ	1273.6	2.91	0	1.9 ± 0.1	1.9 ± 0.2	1.9 ± 0.4	Free
			3.10	0	2.33 ± 0.09	2.57 ± 0.03	2.4 ± 0.2	Bound
141-148	LEEFGDEF	985.4	4.51	0	2.23 ± 0.05	2.64 ± 0.02	3.13 ± 0.05	Free
			4.44	0	2.1 ± 0.2	2.6 ± 0.2	3.3 ± 0.1	Bound
199-208	FVQSGNKPEW	1191.6	3.60	0	3.61 ± 0.07	4.01 ± 0.07	4.32 ± 0.04	Free
			3.68	0	3.8 ± 0.1	4.1 ± 0.1	4.5 ± 0.1	Bound
215-232	PVLPPDLRPLVPLDGGRF	1958.1	4.49	0	8.9 ± 0.1	9.4 ± 0.1	9.5 ± 0.5	Free
			4.36	0	8.4 ± 0.1	8.7 ± 0.09	9.08 ± 0.06	Bound
233-240	ATSDLNDL	848.4	3.61	0	3.3 ± 0.3	3.55 ± 0.08	3.63 ± 0.08	Free
			3.76	0	3.24 ± 0.08	3.43 ± 0.06	3.64 ± 0.08	Bound
260-273	PDIIVRNEKRMLQE	1740.9	3.28	0	5.4 ± 0.1	6.3 ± 0.2	6.2 ± 0.1	Free
			3.41	0	3.0 ± 0.3	3.2 ± 0.6	2.7 ± 0.2	Bound
326-335	VITVGPYLRL	1130.7	4.55	0	1.00 ± 0.08	1.26 ± 0.06	1.5 ± 0.1	Free
			4.41	0	1.6 ± 0.4	1.8 ± 0.3	1.6 ± 0.1	Bound
347-359	ELFKPFIYGKLEL	1596.9	4.69	0	1.59 ± 0.04	2.30 ± 0.06	3.17 ± 0.05	Free
			4.51	0	2.1 ± 0.2	3.1 ± 0.2	3.7 ± 0.2	Bound
379-386	VVWDILDE	988.5	5.00	0	0.99 ± 0.08	1.26 ± 0.06	1.51 ± 0.05	Free
			4.80	0	1.1 ± 0.1	1.49 ± 0.09	1.7 ± 0.1	Bound
387-394	VIREHPVL	962.6	2.98	0	1.23 ± 0.05	1.27 ± 0.04	1.29 ± 0.05	Free
			3.14	0	1.3 ± 0.1	1.3 ± 0.2	1.2 ± 0.1	Bound
414-426	IEGKAIQLHPLVC	1420.8	3.69	0	2.39 ± 0.05	2.35 ± 0.05	2.37 ± 0.04	Free
			3.75	0	2.9 ± 0.2	2.8 ± 0.2	2.48 ± 0.04	Bound
439-446	AVHVPLTL	849.5	3.97	0	0.71 ± 0.06	0.86 ± 0.08	1.0 ± 0.1	Free
			3.99	0	0.9 ± 0.2	0.9 ± 0.2	0.45 ± 0.03	Bound
536-551	VAKTSLKDTTVGRAIL	1673.0	3.35	0	2.33 ± 0.08	2.42 ± 0.09	2.4 ± 0.07	Free
			3.45	0	2.79 ± 0.08	3.0 ± 0.1	3.1 ± 0.09	Bound
581-592	YRILGLKPTVIF	1419.9	4.40	0	2.3 ± 0.02	2.38 ± 0.02	2.31 ± 0.04	Free
			4.29	0	2.3 ± 0.1	2.26 ± 0.04	2.42 ± 0.07	Bound
616-628	MVIPEKKHEIISE	1552.8	2.84	0	3.22 ± 0.07	3.52 ± 0.09	4.0 ± 0.2	Free
			3.03	0	4.5 ± 0.3	4.7 ± 0.3	4.8 ± 0.1	Bound
674-691	QTETVINRDGQEEKQVSF	2108.0	3.28	0	13.8 ± 0.2	15.1 ± 0.1	15.6 ± 0.2	Free
			3.40	0	14.4 ± 0.3	15.3 ± 0.3	16.5 ± 0.2	Bound
716-729	RGLMAKPDGSIIET	1487.8	3.32	0	6.54 ± 0.08	7.45 ± 0.09	8 ± 0.1	Free
			3.76	0	8.1 ± 0.4	8.2 ± 0.1	8.81 ± 0.09	Bound
736-744	REGLNVLQY	1091.6	4.07	0	3.7 ± 0.3	4.1 ± 0.2	4.5 ± 0.3	Free
			4.07	0	3.4 ± 0.2	4.1 ± 0.3	4.6 ± 0.2	Bound
745-760	FISTHGARKGLADTAL	1657.9	3.12	0	4.1 ± 0.2	4.8 ± 0.2	5.5 ± 0.3	Free
			3.25	0	6.3 ± 0.4	6.7 ± 0.4	6.9 ± 0.1	Bound
779-795	LVVTEDDCGTHEGIMMT	1850.8	3.83	0	4.93 ± 0.05	5.2 ± 0.1	5.46 ± 0.03	Free
			3.90	0	5.7 ± 0.2	6.2 ± 0.4	6.04 ± 0.06	Bound
796-802	PVIEGGD	686.3	2.56	0	4.8 ± 0.05	4.82 ± 0.03	4.77 ± 0.05	Free
			2.95	0	4.2 ± 0.1	4.31 ± 0.01	4.54 ± 0.08	Bound
804-818	KEPLRDRVLGRVTAE	1739.0	3.03	0	4.1 ± 0.06	4.5 ± 0.03	4.95 ± 0.05	Free
			3.20	0	5.05 ± 0.02	5.21 ± 0.02	5.3 ± 0.5	Bound
828-836	ILVPRNTLL	1038.7	4.00	0	1.90 ± 0.06	1.95 ± 0.09	2.00 ± 0.07	Free
			4.01	0	2.4 ± 0.1	2.4 ± 0.2	2.1 ± 0.1	Bound
851-864	AVKVRSVVSCDTDF	1525.8	3.56	0	3.6 ± 0.2	3.8 ± 0.1	3.7 ± 0.1	Free
			3.44	0	3.8 ± 0.4	3.8 ± 0.5	4.1 ± 0.8	Bound

875-891	LARGHIINKGEAIGVIA	1732.0	3.18	0	3.2 ± 0.1	3.2 ± 0.4	3.2 ± 0.1	Free
			3.32	0	4.9± 0.1	3.9 ± 0.1	3.6 ± 0.4	Bound
946-956	VITSRNTELKL	1273.7	3.35	0	3.4 ± 0.1	3.5 ± 0.1	3.9 ± 0.2	Free
			3.44	0	3.9 ± 0.1	4.1 ± 0.2	4.2 ± 0.1	Bound
960-975	FGRTKESYKVPYGAVL	1815.0	3.58	0	3.8 ± 0.1	4.2 ± 0.1	4.4 ± 0.2	Free
			3.65	0	4.9 ± 0.2	5.1 ± 0.3	5.2 ± 0.07	Bound
1013-1028	IDGQTITRQTDELTGL	1760.9	4.09	0	11.05 ± 0.04	11.31 ± 0.04	11.39 ± 0.08	Free
			4.11	0	10.3 ± 0.6	10.9 ± 0.2	11.3 ± 0.2	Bound
1060-1070	VLIPGTDMPAQ	1141.6	3.75	0	4.0 ± 0.2	4.2 ± 0.2	4.2 ± 0.3	Free
			3.86	0	4.0 ± 0.3	4.2 ± 0.1	4.6 ± 0.1	Bound
1071-1077	YFLPGKA	795.4	3.52	0	1.44 ± 0.1	1.51 ± 0.11	1.62 ± 0.12	Free
			3.62	0	1.39 ± 0.02	1.53 ± 0.04	1.66 ± 0.05	Bound
1087-1093	ISSGDTL	692.3	3.11	0	2.30 ± 0.01	2.29 ± 0.03	2.2 ± 0.02	Free
			3.34	0	2.18 ± 0.03	2.18 ± 0.04	2.12 ± 0.02	Bound
1117-1128	FEARRPKEPAIL	1426.8	3.13	0	4.4 ± 0.1	4.73 ± 0.09	4.81 ± 0.03	Free
			3.25	0	4.7 ± 0.1	4.9 ± 0.2	5.1 ± 0.2	Bound
1129-1137	AEISGIVSF	922.5	4.48	0	0.72 ± 0.04	0.97 ± 0.02	1.45 ± 0.03	Free
			4.43	0	0.73 ± 0.08	1.1 ± 0.1	1.55 ± 0.08	Bound
1228-1245	IVRQMLRKATIVNAGSSD	1959.1	2.97	0	4.69 ± 0.03	4.88 ± 0.02	4.97 ± 0.05	Free
			3.18	0	5.0 ± 0.2	5.3 ± 0.3	5.4 ± 0.1	Bound
1280-1286	GITKASL	689.4	2.92	0	0.7 ± 0.09	0.8 ± 0.04	0.85 ± 0.04	Free
			3.12	0	1.4 ± 0.2	1.5 ± 0.3	1.0 ± 0.1	Bound
1298-1306	QETTRVLTE	1076.6	2.68	0	2.82 ± 0.04	2.87 ± 0.05	2.89 ± 0.04	Free
			2.98	0	3.5 ± 0.2	3.3 ± 0.2	3.16 ± 0.04	Bound
1308-1321	AVAGKRDELRGLKE	1541.9	1.74	0	2.3 ± 0.1	2.34 ± 0.09	2.3 ± 0.2	Free
			2.14	0	6.7 ± 0.2	6.9 ± 0.2	7.52 ± 0.09	Bound

Expressed as average ± std. error of fit
tr(0): Retention time.

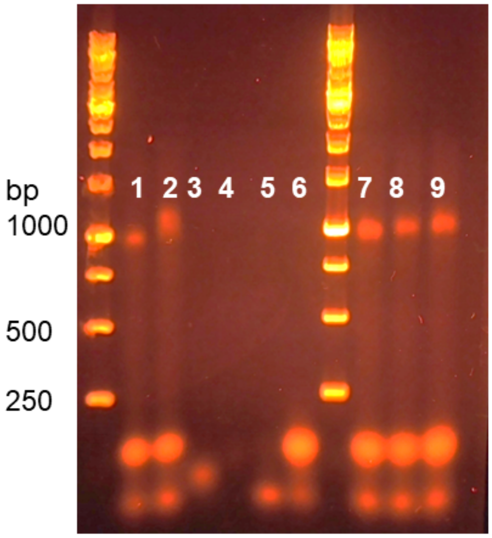

Supplementary Figure S1. Assembly of *ops*-TEC for HDXMS analysis. Free TEC and TEC-bound NusG and RfaH assembly are ascertained by agarose gel electrophoresis (1% agarose gel) as shown in the representative image above. Lanes correspond to: (1) free TEC; (2) TEC-NusG complex; (3) Template (T) DNA; (4) RNA; (5) Non-Template (NT) DNA; (6) T DNA+RNA+NT DNA; (7) free TEC; (8) TEC-RfaH 1:1 complex; (9) TEC-RfaH 1:3 complex.

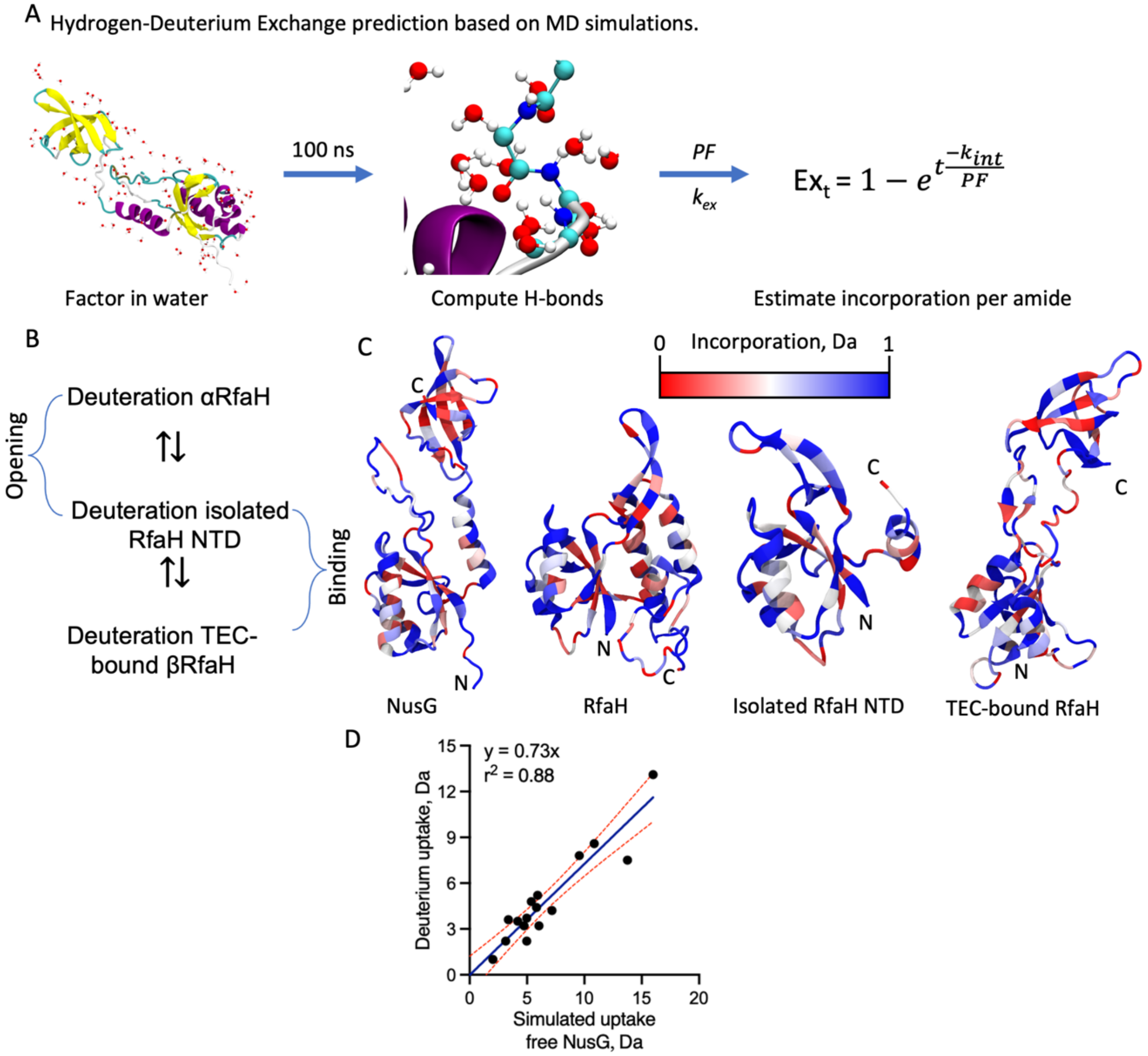

Supplementary Figure S2. Prediction of HDXMS based on MD simulations. A summary of the MD simulation and analysis protocol is shown in (A), where the deuteron incorporation (Ex_t) for each residue is calculated based on their intrinsic exchange constant (k_{int}) and their protection factor (PF) estimated from the partitioning of backbone amide hydrogen bonds between water molecules and the protein. To unveil the effects of interdomain dissociation and binding to the TEC on the experimentally observed deuteron incorporation for RfaH NTD, the changes in predicted deuteration are calculated for the RfaH opening process by comparing full-length RfaH against its isolated NTD and for the TEC binding process by comparing isolated RfaH NTD against TEC-bound RfaH, as shown in (B). The results from the simulation are used to estimate the deuteron incorporation for each residue in NusG, RfaH, the isolated RfaH NTD and the TEC-bound RfaH, as visually presented in (C), where the scale represent the extent of amide incorporation. (D) Correlation between the experimental deuterium uptake of free NusG and the predicted incorporation from MD simulations.

A

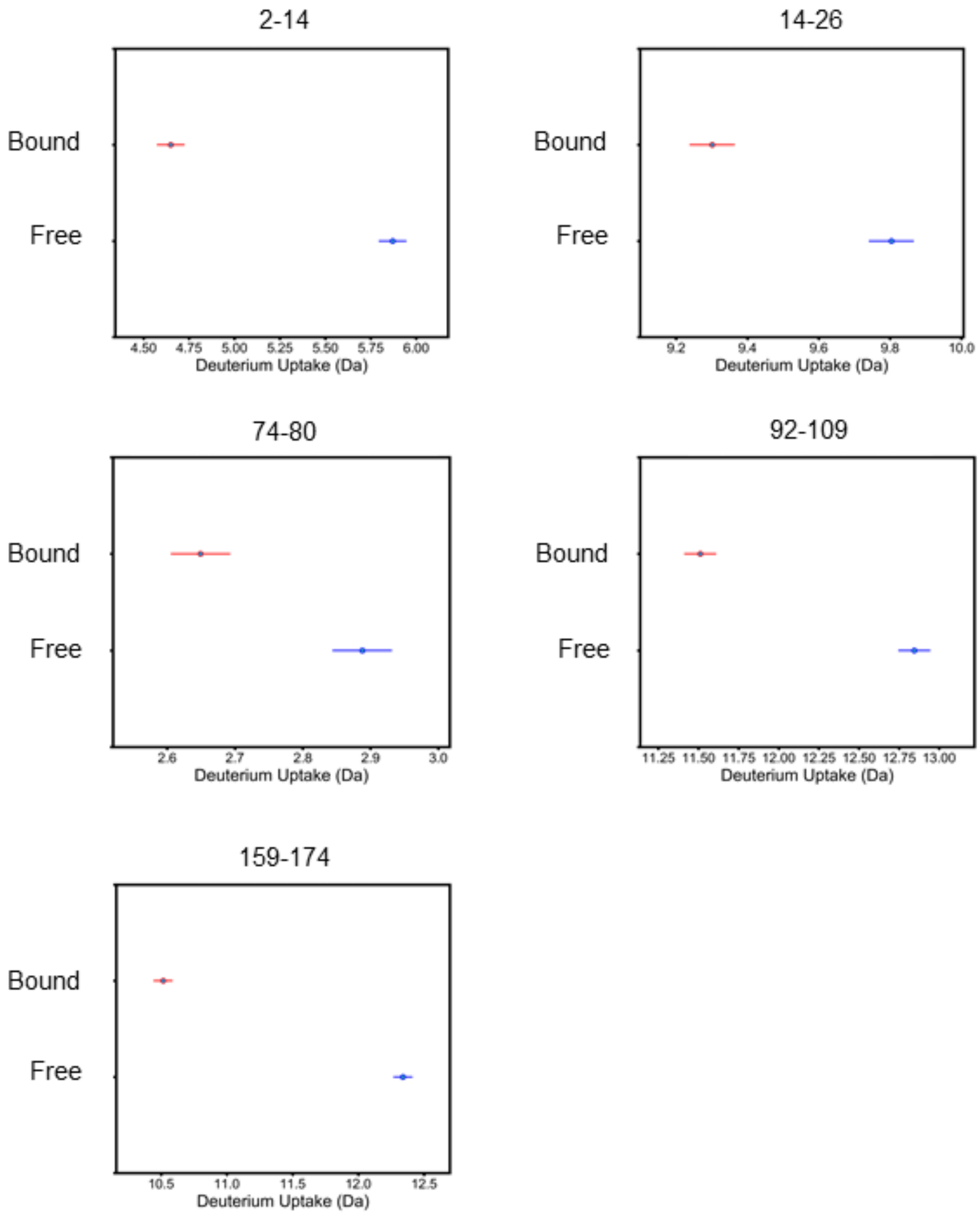

2-14
Bound
Free
4.50 4.75 5.00 5.25 5.50 5.75 6.00
Deuterium Uptake (Da)
14-26
Bound
Free
9.2 9.4 9.6 9.8 10.0
Deuterium Uptake (Da)
74-80
Bound
Free
2.6 2.7 2.8 2.9 3.0
Deuterium Uptake (Da)
92-109
Bound
Free
11.25 11.50 11.75 12.00 12.25 12.50 12.75 13.00
Deuterium Uptake (Da)
159-174
Bound
Free
10.5 11.0 11.5 12.0 12.5
Deuterium Uptake (Da)

B

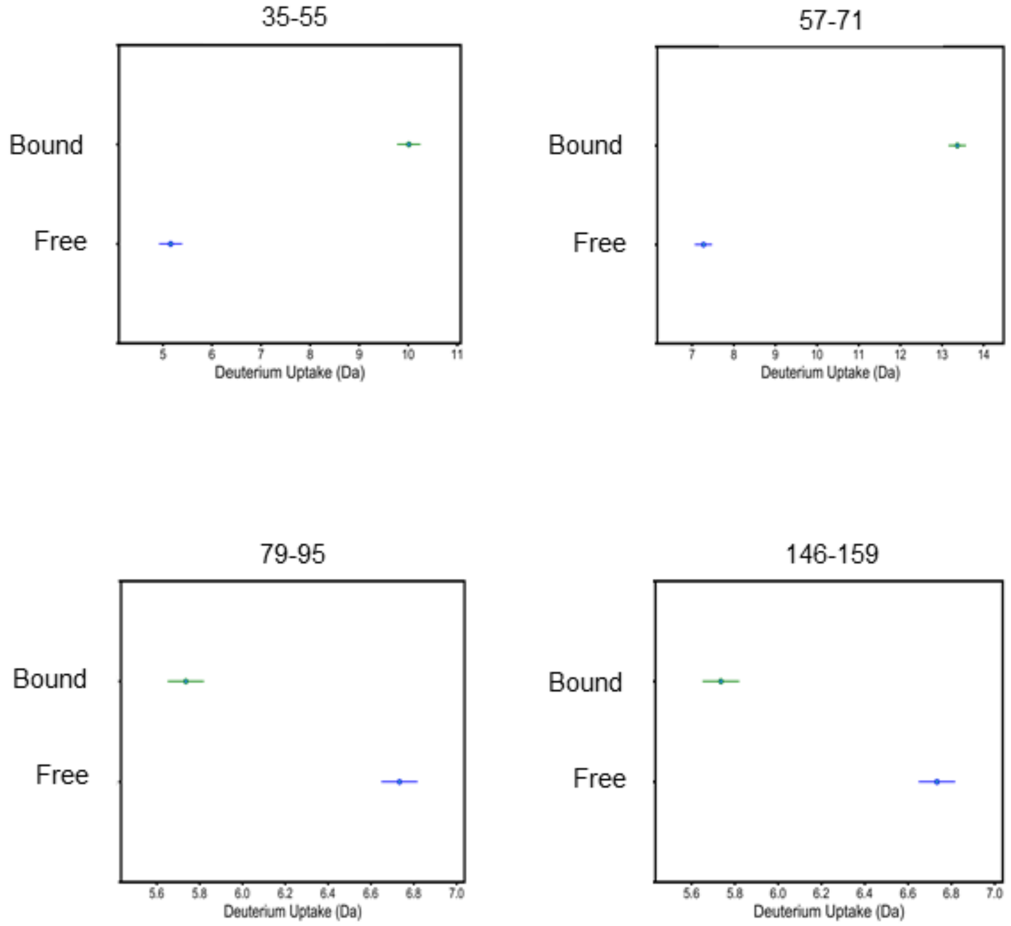
35-55
Bound
Free
5
6
7
8
9
10
11
Deuterium Uptake (Da)
57-71
Bound
Free
7
8
9
10
11
12
13
14
Deuterium Uptake (Da)
79-95
Bound
Free
5.6
5.8
6.0
6.2
6.4
6.6
6.8
7.0
Deuterium Uptake (Da)
146-159
Bound
Free
5.6
5.8
6.0
6.2
6.4
6.6
6.8
7.0
Deuterium Uptake (Da)

C

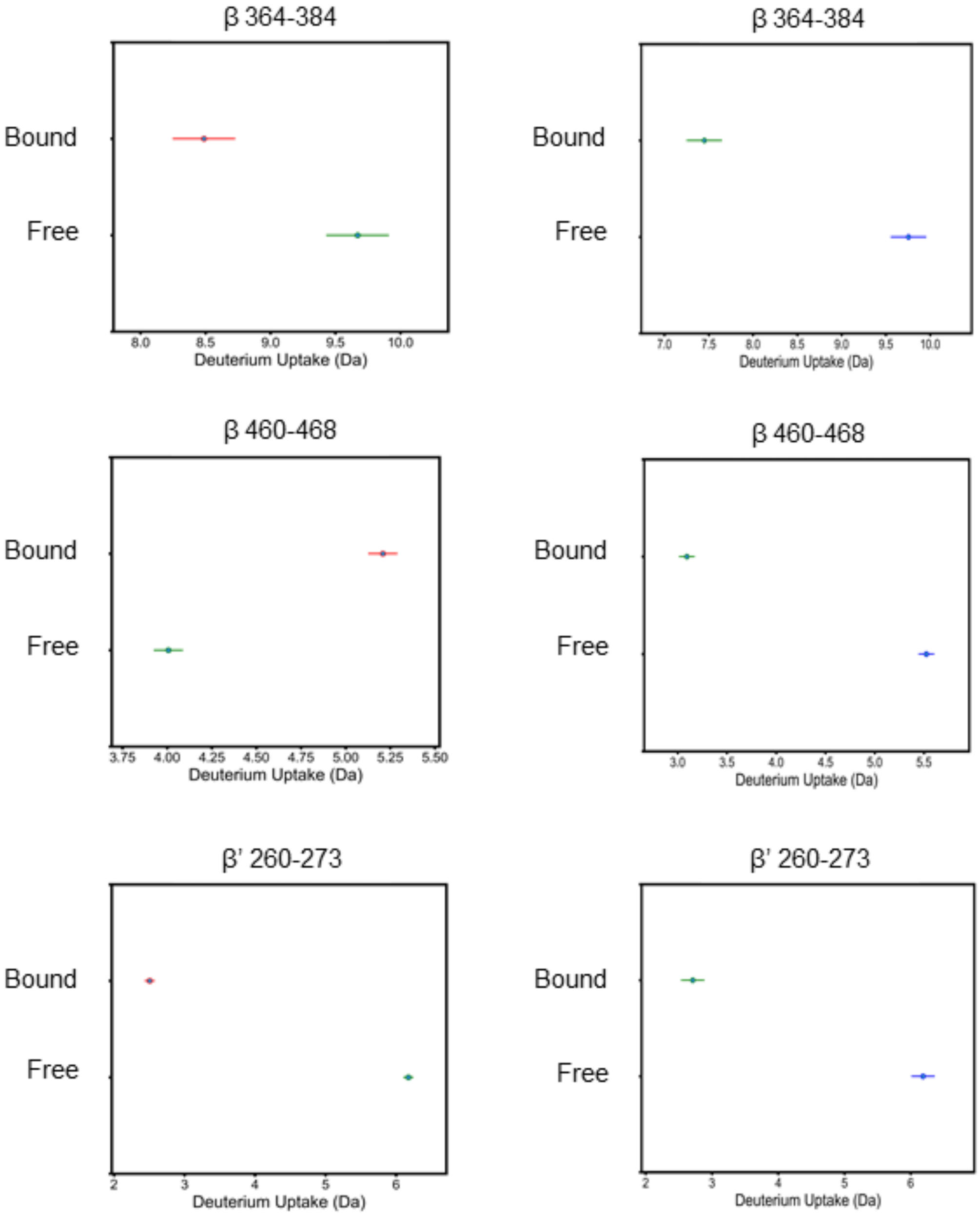

β 364-384
Bound
Free
8.0
8.5
9.0
9.5
10.0
Deuterium Uptake (Da)
β 364-384
Bound
Free
7.0
7.5
8.0
8.5
9.0
9.5
10.0
Deuterium Uptake (Da)
β 460-468
Bound
Free
3.75
4.00
4.25
4.50
4.75
5.00
5.25
5.50
Deuterium Uptake (Da)
β 460-468
Bound
Free
3.0
3.5
4.0
4.5
5.0
5.5
Deuterium Uptake (Da)
β' 260-273
Bound
Free
2
3
4
5
6
Deuterium Uptake (Da)
β' 260-273
Bound
Free
2
3
4
5
6
Deuterium Uptake (Da)

D

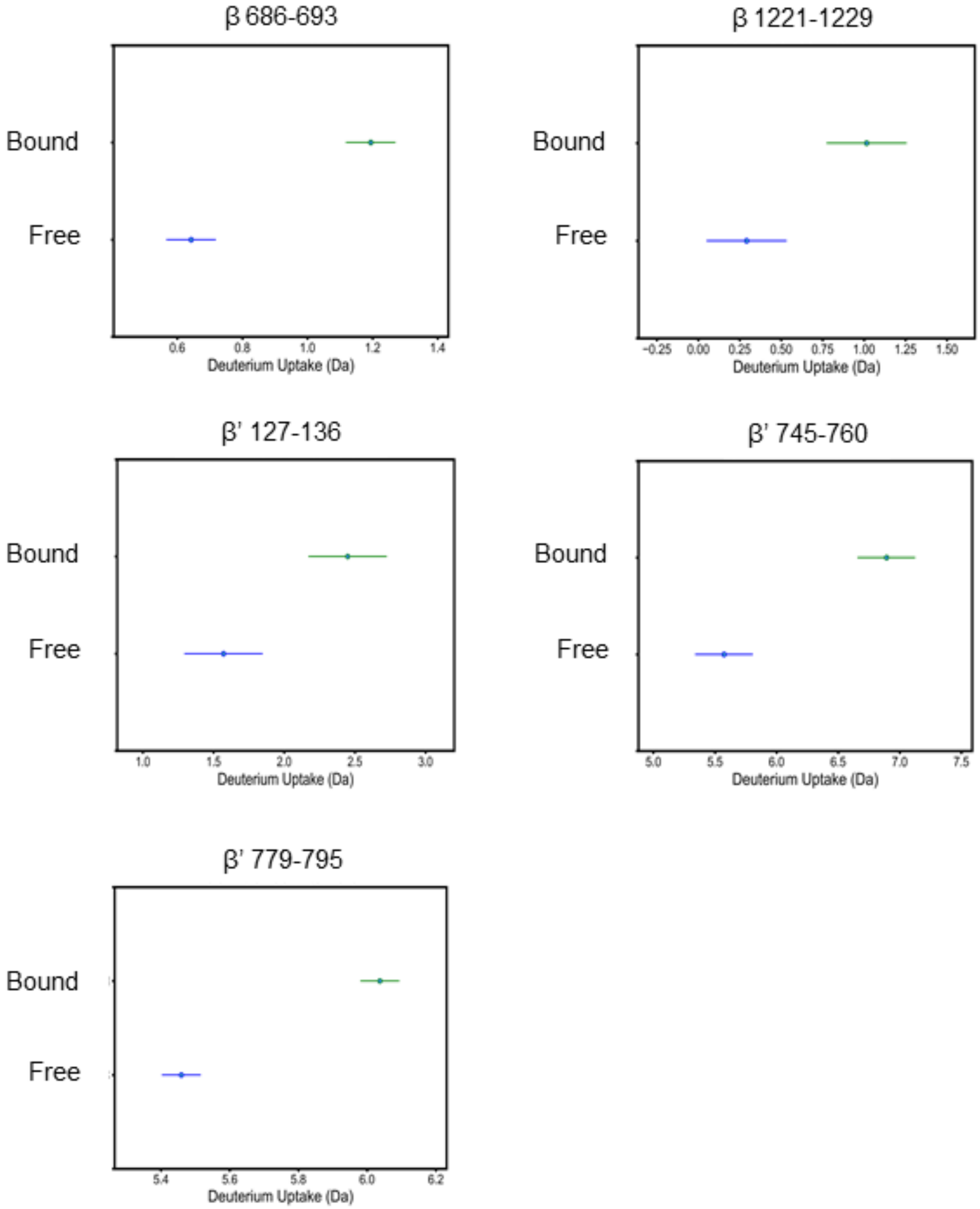

Supplementary Figure S3. Statistically significant differences in deuteron incorporation between free and bound states for different peptides in RfaH (A), NusG (B) and RNAP (C-D). For RNAP, (C) shows changes upon NusG (left) and RfaH (right) binding, whereas changes only ocurring upon RfaH binding are shown in (D). ANOVA analyses and t-tests with a p-value cutoff of 0.05 were implemented in the program DECA to determine the statistical significance of the differences in deuteron uptake for peptides shown in Figures 2, 4, 6 and 7 in the main text. The lines in each plot represent the confidence intervals for each condition, showing that there is no overlap for the observed deuteron uptake differences between conditions.
